# Overcoming Vascular Barriers to Improve the Theranostic Outcomes of Nanomedicines

**DOI:** 10.1002/advs.202103148

**Published:** 2022-03-04

**Authors:** Yufu Tang, Zhongzheng Yu, Xiaomei Lu, Quli Fan, Wei Huang

**Affiliations:** ^1^ Key Laboratory of Flexible Electronics (KLOFE) and Institute of Advanced Materials (IAM) Nanjing Tech University 30 South Puzhu Road Nanjing 211800 P. R. China; ^2^ School of Chemical and Biomedical Engineering Nanyang Technological University Singapore 637459 Singapore; ^3^ Key Laboratory for Organic Electronics and Information Displays and Jiangsu Key Laboratory for Biosensors Institute of Advanced Materials (IAM) Nanjing University of Posts and Telecommunications Nanjing 210023 China; ^4^ Shaanxi Institute of Flexible Electronics (SIFE) Northwestern Polytechnical University (NPU) Xi'an 710072 China

**Keywords:** improved theranostics, nanotheranostic agents, vascular obstacles

## Abstract

Nanotheranostics aims to utilize nanomaterials to prevent, diagnose, and treat diseases to improve the quality of patients’ lives. Blood vessels are responsible to deliver nutrients and oxygen to the whole body, eliminate waste, and provide access for patrolling immune cells for healthy tissues. Meanwhile, they can also nourish disease tissues, spread disease factors or cells into other healthy tissues, and deliver nanotheranostic agents to cover all the regions of a disease tissue. Thus, blood vessels are the first and the most important barrier for highly efficient nanotheranostics. Here, the structure and function of blood vessels are explored and how these characteristics affect nanotheranostics is discussed. Moreover, new mechanisms and related strategies about overcoming vascular obstacles for improved nanotheranostic outcomes are critically summarized, and their merits and demerits of each strategy are analyzed. Moreover, the present challenges to completely exhibit the potential of overcoming vascular barriers to improve the theranostic outcomes of nanomedicines in life science are also discussed. Finally, the future perspective is further discussed.

## Graphical abstract

The information is stratified according to strategies and mechanisms of overcoming vascular obstacles, enabling a comprehensive comparison of these strategies and mechanisms for theranostics of up to different disease positions and disease characters. By summarizing the current research progress, this review may shed light on overcoming vascular obstacles for improved nanotheranostic outcomes.

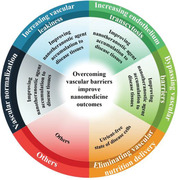

## 1 Introduction

**Figure 1:**
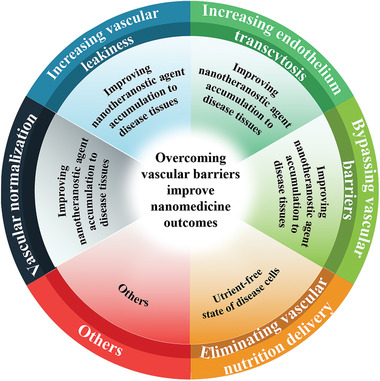
The general orientation of overcoming vascular obstacles for improved nanotheranostic outcomes.

In the field of medicine, nanotheranostic agents with tunable pharmacokinetics, biodistribution, and multifunctionality hold great promise for a more personalized theranostic approach compared with conventional small molecule agents.^[^ ^1^, ^2^, ^3^, ^4^, ^5^ ^]^ Nanotheranostics for different types of diseases aim to utilize nanomaterials to prevent, diagnose, and treat diseases for a better understanding of the complex and dynamic pathologic microenvironment, and to improve the quality of patients’ lives. This concept has driven the development of nanomedicine in the past three decades. Currently, only few nanotheranostic agents, such as pegylated liposomal doxorubicin (Doxil), have been approved for clinical applications.^[^ ^6^ ^]^ Moreover, most of these clinically approved agents can only mitigate adverse effects but cannot or only modestly improve theranostic efficacies.^[^ ^6^ ^]^ The reason is that pathophysiological barriers will prevent these nanomedicines from realizing their theranostic potential.^[^ ^2^, ^7^, ^8^, ^9^, ^10^ ^]^ Among these pathophysiological barriers, vasculature obstacle is the first and the most important barrier.^[^ ^11^ ^]^ Blood vessels are responsible for eliminating waste, delivering nutrients and oxygen to the whole body, and providing accesses for patrolling immune cells. The diffusion rang of oxygen from vessels to the surrounding tissue cells is limited 100 to 200 µm. Thus, tissue exceeding this size range can only grow by vasculogenesis and angiogenesis to form new blood vessels. Similarly, theranostic agents are difficult to be delivered to cover all the regions of a disease tissue in effective quantities without an efficient blood supply. However, blood vessels also nourish disease tissues, such as cancer—a life‐threatening major disease. For example, without adequate angiogenesis, tumor will not grow to a diameter over 0.4 mm and cannot cause tumor metastasis.^[^ ^12^, ^13^ ^]^ Based on the specific role and dual functions of blood vessels, scientists have currently provided five general orientations to overcome vasculature obstacle for efficient delivery of nanotheranostic agents (**Figure** **1**).

The first general orientation is to increase vascular endothelium permeability, which increases the ratio of nanotheranostic agents passing across vascular walls and arriving at a large fraction (close to 100%) of disease cells. In 1998, Jain's group reported the existence of large permeable gaps with a size range up to 2000 nm between endothelial cells of mouse tumor models. While the gaps between vascular endothelial cells in healthy tissues are too small (≈2–6 nm).^[^ ^5^ ^]^ Subsequently, the existence of native large permeable gaps between the blood vessels of other disease tissues, such as inflammation, age‐related macular degeneration, diabetic retinopathy, “abnormalization” of the vasculature skin psoriasis, neurodegenerative diseases, rheumatoid arthritis and neuropathic pain models, hemangiomata, collagen synthesis diseases,^[^ ^14^ ^]^ was also observed.^[^ ^15^ ^]^ Scientists use these findings as a rationale to design nanotheranostic agents within the gap size range of disease tissues and healthy tissues, expecting them to passively enter diseased tissues but not healthy tissues. In fact, the ratio of nanotheranostic agents reaching disease tissues is very low. For example, only 0.7% theranostic agents can reach the solid tumors due to the vascular obstacles,^[^ ^16^ ^]^ indicating that native permeable gaps of tumor vessels are insufficient to implement high theranostic outcomes. However, Leong and co‐workers found some specific nanoparticles with tunable diameter, density, charge and dose could induce size‐, time‐, and space‐different endothelial leakiness,^[^ ^11^, ^17^ ^]^ which further innovated this concept of native permeable gaps. Additionally, Setyawati and Leong also demonstrated some frequently used theranostic nanoparticles, such as mesoporous silica nanoparticles^[^ ^18^ ^]^ and gold nanoparticles,^[^ ^19^ ^]^ can be applied as antiangiogenesis agents in a conventional drug‐free manner to induce tumor vessel normalization. In fact, vessel normalization also improves the delivery efficiency of nanotheranostic agents.^[^ ^20^, ^21^ ^]^ Thus, some observed high accumulation of theranostic agents in disease tissues may be caused by the vessel leakiness or normalization induced by nanotheranostic agents themselves rather than the native permeable gap effect.^[^ ^18^ ^]^ In 2020, Chan and co‐workers also innovated the concept of native permeable gaps. They found that only a very low frequency of gaps between endothelial cells was observed in various tumor types. Overall, only 26 gaps were found in the 313 blood vessels of the tumor models.^[^ ^5^ ^]^ The overall gap coverage was just 0.048% of the vascular surface area. Surprisingly, only 7 of the 26 gaps were interendothelial, while the other 19 gaps were transcellular channels. The ratios of accumulated 15 nm, 50 and 100 nm PEGylated gold nanoparticles completely caused by the native large permeable gaps of the tumor blood vessels were 12%, 3%, and 25%, compared with the control, respectively. Thus, the relative contribution of native gaps of tumor for nanoparticles is unpredictable and variable. In fact, they further demonstrated that up to 97% of nanoparticles could get into tumors via an active transcytosis of vascular endothelial cells.^[^ ^5^ ^]^

The second general orientation is vascular endothelium transcytosis, which increases the ratio of nanotheranostic agents passing across vascular walls and arriving at a large fraction (close to 100%) of disease cells. Nicolae Simionescu, in 1979, first created the concept of transcytosis of macromolecules and coined mechanisms of the endothelial cell vesicles to describe the bidirectional transports of macromolecules between the blood and the tissues.^[^ ^22^ ^]^ The main vesicular structure in endothelial cells for this function are caveolae, ensuing from the cholesterol‐rich membrane domains, called lipid rafts, induced by binding of ligands to specific endothelial transmembrane glycoproteins including APP and PV1.^[^ ^22^, ^23^ ^]^ Transcytosis includes receptor‐mediated transcytosis (the specific ligand–receptor recognition for transcytosis), absorption‐mediated transcytosis (unspecific absorption on cellular membranes) and fluid‐phase transcytosis (without interacting with cellular components and in a dissolved form in the lumen of vesicles).^[^ ^22^, ^24^, ^25^ ^]^ Transcytosis pathway has long been seen as an effective route to overcome vascular obstacles for nanomedicines, but its efficiency in clinical exploitation is still unknow.^[^ ^26^ ^]^

The third general orientation is to reduce or eliminate nutrition delivery to the disease tissues via vasculature. Since blood vessels also nourish disease tissues, selectively reducing or eliminating vascular nutrient delivery to disease cells by nanotheranostic agents will lead to a nutrient‐free state of disease cells, which eventually starves the disease cells to death. Frequently used methods include reduction and/or embolization of existing blood vessel and/or inhibition of new vessel formation of disease tissues. For patients who exhibit a poor response to chemotherapy or have recurrent or unresectable cancer and other angiogenic diseases, such strategy might be a good method as an alternative therapy. Currently, the US Food and Drug Administration has approved the use of some therapeutic agents utilizing this strategy, such as vascular endothelial growth factor (VEGF)‐specific antibody bevacizumab (Avastin; Roche/Genentech), sorafenib (Nexavar; Bayer), sunitinib (Sutent; Pfizer), pazopanib (Votrient; GlaxoSmithKline), and vandetanib (Zactima; AstraZeneca).^[^ ^15^ ^]^ Although promising, clinical research demonstrated that antiangiogenic therapy exhibits short or slight therapy outcomes, and cancer recurrence. In addition, a long‐term use is faced with the problem of drug resistance.^[^ ^27^ ^]^

The fourth general orientation is vessel normalization. With reverse thinking, normal vasculature is easier to deliver oxygen and nutrients to the required parts of human body than that of the abnormal vasculature, Jain proposed a concept that “normalizing” the abnormal blood vessels in cancer and other angiogenic diseases could improve the delivery and effectiveness of nanotheranostic agents, rising as a complementary therapeutic paradigm for cancer and other vascular diseases.^[^ ^20^, ^21^ ^]^ These opinions bear the embryo of a new and right mechanism, as well as the motivation for the development of different mechanisms. With new insights into in vivo nanotheranostic agent across the blood vessels, we should rethink our strategies by shifting our focus from only considering nanotheranostic agents for passive native permeable gaps delivery to designing nanotheranostic agents for improved vessel extravasation.

The fifth general orientation is bypassing vascular obstacles. Some vascular obstacles are difficult to overcome through the above general orientations. Even if obstacles were overcome, it could also cause severe side‐effects. Thus, bypassing vascular obstacles to improve nanotheranostic agent delivery to disease tissues is very popular. Some specific administration routes are developed, such as intranasal administration. However, such strategy is only suitable for overcoming vascular obstacles of specific tissues, mainly used for blood‐brain barrier (BBB).

During the last two years, new mechanisms and related works about overcoming vascular obstacles are popping up. These insights pave approaches to overcome vascular obstacles for improved nanotheranostic outcomes and encourage scientists to rethink how diseases can be treated, the effect is far from satisfactory. Now is the right time to critically review the development process of overcoming vascular obstacles for improving nanotheranostic outcomes, and to present detailed mechanisms of each strategy with analysis of their merits and demerits. Although several excellent reviews made partly related topics, these papers are either focused on specific tumor vessels or emphasized on the specific theranostic nanomaterials. However, we stratify the information according to strategies and mechanisms of overcoming vascular obstacles, enabling a comprehensive comparison of these strategies and mechanisms for theranostics of up to different disease positions and disease characters (**Table** **1**). Increased awareness and consideration of overcoming vascular obstacle properties will contribute to the development of more effective nanotheranostic strategies. Additionally, the structure and function of blood vessels is explored and how these characteristics affect nanotheranostics is discussed. A future outlook of this fast‐growing research field will also be discussed. By summarizing the current research progress, this review may shed light on overcoming vascular obstacles for improved nanotheranostic outcomes, while we hope to trigger continuous breakthrough in this realm.

**Table 1:** Summary of the general orientations of overcoming vascular obstacles for improved nanotheranostic outcomes

The general orientations of overcoming vascular obstacles for improved nanotheranostic outcomes	Advantages	Challenges
Improved nanotheranostic agent delivery to disease tissues by increasing endothelium leakiness	Suitable for almost all diseases	Balancing theranostic effect and side‐effect, excessive endothelium leakiness also increases the possibility of malposition of nutrients, functional biomolecules and cells on either side of the vessel wall. Off‐target‐caused serious side effects, such as hemorrhage, hypertension, thrombus, clots in the arteries and gastrointestinal perforations.
Improved nanotheranostic agent delivery to disease tissues by increasing endothelium transcytosis	High efficiency	One of the crucial questions of the first step is that endocytosis agent transcytosis rather than degradation pathway. This step determines the whereabouts of endocytosed theranostic agents: 1) recycling back to the plasma membrane, degradation by trafficking into lysosomes, and 2) exocytosis by the Golgi apparatus.
Improved nanotheranostic agent delivery to disease tissues by bypassing vascular obstacles	High efficiency	Only overcoming of vascular obstacles in specific tissues, mainly for BBB.
Selectively reduce or eliminate nutrition delivery to the disease tissues via vasculature (reducing and/or embolizing existing blood vessel and/or inhibiting new vessel formation of disease tissues)	Prevent cancers from becoming more malignant and metastatic, and to increase the responsiveness to chemotherapy, immunotherapy and radiation therapy.	This strategy can only be eradicated disease completely in combination with other theranostic model. For example, agent‐induced vascular thrombosis in the tumor tissues cannot cause long‐term tumor eradication because tumor cells of the surviving rim of the tumor can still be nourished by adjacent non‐tumoral blood vessels and tissues, resulting in tumor regrowth; Off‐target side effects including increased risk of arterial thromboembolic events. The treatment is also related to a number of adverse events, such as causing tumor hypoxia, recruiting proangiogenic and proinflammatory stromal cells and decreasing chemotherapeutic drug penetration.
Improved nanotheranostic agent delivery to disease tissues by vascular normalization	Preventing cancers from changing into more malignant and metastatic; increasing the responsiveness to chemo‐, immune‐, and radiation‐therapy	Vessel normalization occurs transiently during a normalization window, general 6 days. Owing to narrow normalization window, it needs to carefully control the dosage and dosing period of antiangiogenic drug to adjust angiogenic and antiangiogenic balance for the appearance of vessel normalization. Thus, the strategy of vessel normalization is still challenging in clinic.
Others (such as transient stealth coating of liver sinusoidal wall)	A generic strategy for almost all nanotheranostic agents	Immature theoretical mechanism; unknown long‐term side‐effect.

## 2 Nanotheranostic Agents

**Figure 2:**
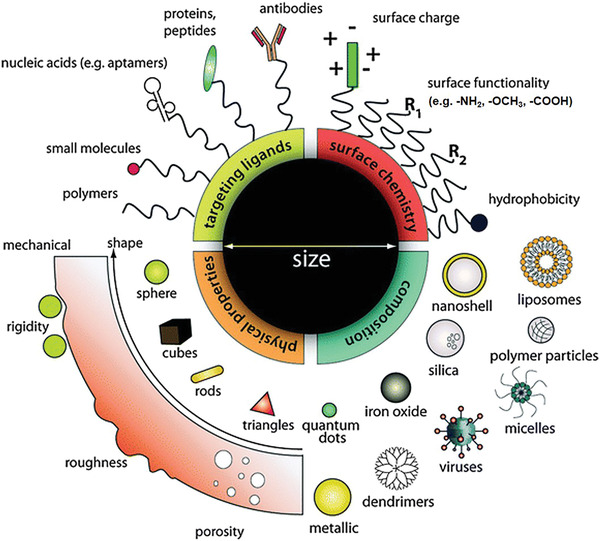
Schematic illustration of typical and multifunctional nanotheranostic agent formulations. Reproduced with permission.^[^ ^28^ ^]^ Copyright 2011, Royal Society of Chemistry.

Materials in the nanoscale range will have larger surface areas, which has shown great potential in the ease of multifunctionality, extremely attracting in recent decades. Many different types of nanomaterials (**Figure** **2**),^[^ ^28^ ^]^ such as organic materials (including liposomes, polymers, dendrimers, and micelles),^[^ ^29^, ^31^ ^]^ inorganic materials (including silica, iron oxide, metallic, carbon nanotubes, and fullerenes),^[^ ^32^, ^33^, ^34^, ^35^, ^36^ ^]^ and organic and inorganic hybrid materials (including metal–organic frameworks), have been used for nanotheranostic agents in medical applications. Nanotheranostic materials generally include the following constituents: targeting moieties (for specific recognition of target sites), theranostic moieties (for theranostic function), and biocompatible and water‐soluble moieties. Additionally, the surface and shape of nanomaterials can be modulated according to requirements. Ideally, after the completion of theranostic functions, the residual nanotheranostic agents will be safely degraded or excreted from the body.^[^ ^4^ ^]^ Some nanotheranostic agents, such as pegylated liposomal doxorubicin (Doxil), have been critically reviewed.

## 3 The Role of Blood Vessel in Nanotheranostics

### 3.1 The Function and Structure of Blood Vessel

**Figure 3:**
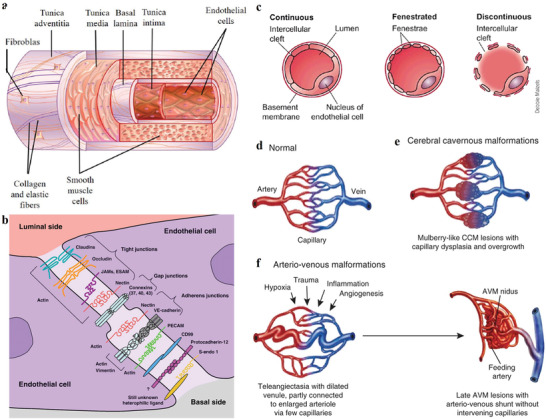
a) Histological structure of a blood vessel. Reproduced with permission.^[^ ^38^ ^]^ Copyright 2018, Whioce Publishing Pte. Ltd. b) Scheme of a protein structure of endothelial intercellular junctions. Reproduced with permission.^[^ ^40^ ^]^ Copyright 2008, Elsevier. c) Different cellular morphology of capillaries. Reproduced with permission.^[^ ^41^ ^]^ Copyright 2008, Springer Nature. d–f) Cellular mechanisms of cerebrovascular malformations. Reproduced with permission.^[^ ^42^ ^]^ Copyright 2011, Springer Nature.

Blood vessels are the channels or conduits that deliver blood, nutrients and oxygen to various sites of the whole body. The blood vessels include two closed systems of tubes that they are all the beginning and end of the heart. One of systems is the pulmonary vessels, which is responsible for the blood circulation from the right ventricle to the lungs and then to the left atrium. The other system is the systemic vessels, which is responsible for the blood circulation from the left ventricle to various sites of whole‐body tissues and then to the right atrium. Blood vessels are classified as arteries, capillaries, and veins according their structures and functions. In healthy body, vasculature systems not only transport body essentials, such as blood cells, nutrients, and oxygen, to the organ tissues, but also take non‐necessity, such as toxin, waste, and carbon dioxide, away from the body. In the process of disease theranostics, most of theranostic agents are administered into the human body and are delivered via the bloodstream to reach the surrounding tissue of disease cells by frequently used intravenous, oral, pulmonary, and transdermal routes. Therefore, their theranostic ability is strongly dependent on the structure and function of vessels and transport properties of theranostic agents in tissue.^[^ ^37^, ^38^ ^]^ Blood vessels include three generic layers (**Figure** **3**a),^[^ ^38^, ^39^ ^]^ Fibroblasts, vasa‐vasorum, proteoglycans, and collagen. They are separated from the media by the external elastic lamina, make up the outer layer of blood vessels, also called adventitia. Collagen fibers and smooth muscle cells that is separated from the intima by the inner elastic lamina constitute the intermediate layer. Underlying connective tissue and endothelial cells compose the intima. The vascular endothelium, which is composed of endothelial cells of a homogeneous, continuous and tight monolayer, and cobblestone morphology, covers a surface area of almost 1000 m^2^.^[^ ^39^ ^]^ As one of the largest organs in the body, the endothelium is associated with many physiological functions and pathological process, such as regulation of cellular and nutrient trafficking as well as vasomotor tone, maintenance of blood fluidity as well as the local balance of proinflammatory and anti‐inflammatory mediators, and generation of new vessels.

The endothelial junction, including tight junctions, gap junctions, and adherens junctions, based on a complex set of junctional proteins is one of elements for the endothelial barrier (Figure 3b).^[^ ^40^ ^]^ Tight junctions seal the intercellular cleft of adjacent endothelial cells, and adherens junctions will provide the mechanical strength of opposing cells, gap junctions form connexin‐mediated transmembrane channels between neighboring cells that allow ions to pass, water and also other small molecules.

### 3.2 The Endothelial Heterogeneity of Blood Vessel In Vivo

The morphological and functional heterogeneity of endothelium in vivo is mainly reflected in vessel size‐, organ‐, and age‐specific differences. Additionally, the blood vessels of disease tissues also exhibit obvious differences compared with normal tissues.

Morphological heterogeneity of vascular endothelium is most obvious. According to the frequently used classification method, endothelial cell vessel phenotype can be classified as continuous, fenestrated or discontinuous (Figure 3c).^[^ ^41^, ^42^, ^43^ ^]^ Most obviously, different organs and tissues exhibit significant morphological heterogeneity. For example, endothelial cells of large arteries are often polygonal, nonfenestrated and thicker, while endothelial cells of microvessels are usually flattened, elongated, and fenestrated. There are even some organ‐specific differences among capillary beds. Continuous endothelium can be observed in most arteries, veins and capillaries of the brain, skin, lungs, and heart (**Table** **2**).^[^ ^44^ ^]^ Fenestrated endothelium is found in choroids plexus, gastrointestinal tract, endocrine and exocrine glands, kidney glomeruli, and a subgroup of renal tubules. And beyond morphological heterogeneity, another obvious and vital heterogeneity of endothelial cells is their functional heterogeneity, such as their roles in the control of vasoconstriction and vasodilation, leukocyte homing and diapedesis, blood coagulation and anticoagulation, fibrinolysis, antigen presentation, acute inflammation and wound healing, atherogenesis, and catabolism of lipoproteins. Additionally, endothelial cells of different organ tissues also show functional heterogeneity. For example, brain endothelial cells can form a continuous endothelium by complex tight junctions with astrocyte end feet, which produce the BBB to highly regulate polarized endocytosis and transcytosis. By contrast, fenestrated and discontinuous liver endothelial cells line the hepatic sinusoids to control the processing of toxins and the exchange of metabolites between the portal blood, hepatocytes, and Kupffer cells.

**Table 2:** Types of blood vessels in various organs with different permeability.^[^ ^47^ ^]^

Vasculature types		Endothelial cell types	Interendothelial junctions	Typical organs	Estimated upper limit for paracellular transportation
Continuous type (no fenestrae)	Continuous basement membrane	No fenestrae type	Tight junctions and adherens junctions	Retina, spinal cord, brain, thymus	Determined by interendothelial junctions (tight junctions) <1 nm
			Adherens junctions with limited contribution of tight junctions	Skin, heart, muscle, lung, adipose tissue,	Determined by interendothelial junctions (adherens junctions) <5 nm
Fenestrated type		Fenestrated type (with diaphragm)		Skin, exocrine glands, endocrine glands, intestinal mucosa, kidney (peritubular), lymph node	Determined by diaphragm <6–12 nm
		Fenestrated type (open pores without diaphragm)		Kidney (glomerulus)	Determined by glycocalyx <15 nm
Discontinuous type (sinusoidal)	Discontinuous basement membrane	Fenestrated type (with and/or without diaphragm)		Liver, spleen	<50–280 nm, largely differ among species <3–5 µm

### 3.3 The Vascular Barriers for Nanotheranostics

Continuous endothelium monolayer of blood vessel is the major barrier of delivering theranostic agents and nutrition to the surrounding tissue of disease cells. The permeability of blood vessels involves size barrier and charge barrier. The luminal glycocalyx layer with negative surface charge of endothelial cells constitutes the “charge barrier” of negatively charged nanotheranostic particles due to electrostatic repulsion. The interendothelial paracellular permeability, dominating the size barrier, is regulated by the interendothelial tight junctions and/or adherens junctions. In healthy tissues, the hydrophilic channel between two adjacent endothelial cells of the tightest central nervous system endothelium (BBB) is ≈0.8 nm.^[^ ^44^ ^]^ The typical width of intercellular gaps on the nonleaky microvascular endothelial cell barrier is 10–25 nm.^[^ ^45^ ^]^ Additionally, the transcellular pathway may dominate the paracellular pathway for the vascular permeability of selective molecules, especially in vessels with tight interendothelial junctions. Different with the blood vessel of normal tissues (Figure 3d),^[^ ^42^ ^]^ the many diseases, such as diabetic retinopathy, inflammation, and cancer, are characterized by highly abnormal, defective, hypervascular networks, consisting of immature, leaky, and irregular vessels with a marked loss of perivascular cell coverage (Figure 3e,f).^[^ ^42^ ^]^ In particular, these characteristics in the blood vessels of the tumor are highlighted because quick multiplication and division of tumor cell induce more angiogenesis to provide them with plenty of nutrients. The pore size in the leaky tumor vasculatures has been reported to range from 380 to 780 nm.^[^ ^44^ ^]^ Although most of the blood vessel of tumor tissues exhibit leakage, the nanotheranostic agents are difficult to pass the blood vessels due to high interstitial fluid pressure in a solid tumor.^[^ ^46^ ^,47]^ Additionally, the blood vessels of some disease tissues are highly abnormal and defective, nanotheranostic agents are difficult to be delivered to cover all the regions of a disease tissue in effective quantities without an efficient blood supply.^[^ ^37^ ^]^

## 4 Strategies to Overcome Vascular Obstacles to Improve Nanotheranostic Outcomes by Nanotheranostic Agents

### 4.1 Increasing Vascular Endothelial Transcytosis to Overcome Vascular Obstacles

Transcytosis, an adenosine triphosphate‐mediated active transport process, includes three steps:^[^ ^48^ ^]^ first, vesicles on one side of the cells endocytose or internalize nanotheranostic agents; second, vesicles including nanotheranostic agent are intracellularly trafficked; third, nanotheranostic agents are exocytosed or released to the other side of the cells. So the first step is the determined step of three destinies of endocytosed nanotheranostic agents. The first destiny: vesicles including nanotheranostic agents are recycled back to the plasma membrane again; the second destiny: vesicles including nanotheranostic agents are trafficked into lysosomes to be degraded; the third destiny: vesicles including nanotheranostic agents are exocytosis by the Golgi apparatus. The crucial question of the first step is that endocytosis mediates transcytosis rather than degradation pathway, which will determine the efficiency of transcytosis. Caveolae‐mediated endocytosis or micropinocytosis of agents ordinarily leads to a low probability of lysosomal degradation but mainly leads to exocytosis of mitochondria or Golgi apparatus. Caveolae bud from the plasma membrane in a dynamin and guanosine triphosphate‐dependent manner can form caveolin‐coated, omega‐shaped plasmalemmal invaginations with a diameter of 60–70 nm.^[^ ^49^, ^50^ ^]^ Thus, increasing transcytosis of nanotheranostic agents can effectively augment nanotheranostic agents to cross vascular obstacles to access disease tissues to improve nanotheranostic outcomes. Transcytosis includes receptor‐mediated transcytosis, absorption‐mediated transcytosis, and fluid‐phase transcytosis. In 2020, Chan and co‐workers found that up to 97% of nanoparticles pass cross the blood vessel to enter tumor tissues through an active process of vascular endothelial cells, which indicated that native permeable gaps of endothelial cells in tumor are not main route for the nanoparticles to pass across vascular wall and enter solid tumors.^[^ ^5^ ^]^

#### 4.1.1 Ligand‐Mediated Increase in Vascular Endothelial Transcytosis

Vascular endothelial cells in disease tissues overexpress a series of biomarkers provide chances to develop actively targeted nanotheranostic agents with ligands that specifically recognize and bind to these biomarkers. Generally, transcytosis of vascular endothelial cells is initiated by fast endocytosis via the surface interactive electrostatic adherence of nanotheranostic agents. Ligands, such as peptide, glucose, transferrin and albumin affinity peptides, proteins, aptamers, and antibodies, can be chemically arrayed on the surface of nanotheranostic agents to enhance cell uptake.

**Figure 4:**
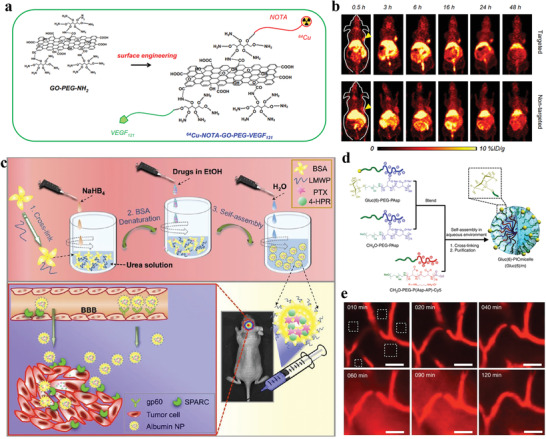
a) The surface engineering of graphene oxide (GO) nanotheranostic agents. b) In vivo VEGFR targeted (top) and nontargeted (down) PET imaging of U87MG tumor‐bearing mice at different time points. (a,b) Reproduced with permission.^[^ ^51^ ^]^ Copyright 2015, Elsevier. c) Synthesis route and the mechanisms of targeted albumin nanoparticles. Reproduced with permission.^[^ ^65^ ^]^ Copyright 2016, American Chemical Society. d) Illustration of oppositely charged block copolymers self‐assembly into Gluc(6)/m. e) Sequential Gluc(6)/m images of mice brain. After a 24 h fast, the 25% Gluc(6)/m (red fluorescence signal) were intravenously injected to a mouse, followed by an intraperitoneal injection of 20 wt% glucose 30 min later. d,e) Reproduced with permission.^[^ ^66^ ^]^ Copyright 2017, Springer Nature.

In 2015, Cai and co‐workers demonstrated that VEGF_121_‐functionalized graphene oxide (GO) nanoconjugates obviously improved vascular targeting efficacy of in vivo tumor due to the targeting of VEGF_121_ ligand (**Figure** **4**a), which significantly enhances tumor accumulation (>8%ID g^−1^), exhibiting great potential for improved tumor nanotheranostic outcome (Figure 4b).^[^ ^51^ ^]^ The theranostics of brain disease suffers from a general limitation that nanotheranostic agents are “blocked” to pass cross the vascular wall to enter into the brain due to BBB, Additionally, blood‐brain tumor barrier is another important limitation for brain tumor theranostics.^[^ ^52^ ^]^

Additionally, endothelial cells of diverse organs and disease tissues exhibit different types of physiological and pathological characteristics. Based on these differences, researchers developed a versatile and controlled vascular immunotargeting strategy to precisely enhance localization in vascular areas of interest and control addressing of the nanotheranostic agents through using antibodies, antibody fragments and other ligands with specific affinity to endothelial surface determinants.^[^ ^53^, ^54^ ^]^ These endothelial surface determinants include angiotensin‐converting enzyme, cell adhesion molecules (CAMs), caveolar determinants (PV1 and APP), and other determinants.^[^ ^55^, ^56^ ^]^ Among them, ligands of PV1 and caveolar APP are used to deliver nanotheranostic agents into caveolae. Intercellular adhesion molecule 1 (ICAM‐1) can effectively target to pathological endothelium. Ligands of platelet‐endothelial cell adhesion molecule (PECAM) deliver drugs and carriers to PECAM constitutively expressed on endothelium throughout the vasculature.^[^ ^55^ ^]^ Their enhanced delivery mechanisms include natural endocytic pathways or uncanonical internalization mechanisms induced by clustering target molecules allows reaching the intracellular vesicles, cytosol or transcellular transport, which provides varieties of surface retention, internalization, and trafficking of drugs and carriers targeted to endothelial determinants. Recently, to address the challenge to deliver drugs into brain, ligands of VCAM‐1 were used to conjugated with nanocarriers for increasing the cerebral accumulation. The results indicated that the uptake of anti‐VCAM is >10‐fold greater than antibodies to transferrin receptor‐1 and ICAM‐1 in the inflamed brain, which provides platform to target inflamed brain vasculature, thus benefiting numerous brain pathologies.^[^ ^57^ ^]^ Additionally, many theranostic agents, such as nucleic acids, enzymes, and genes, must be deliver into cells to play their part.^[^ ^58^ ^]^ For example, vascular immunotargeting of antioxidant enzymes, such as endothelial superoxide dismutase, plays an important role in multiple vascular functions, including high effectiveness and specificity of interception of ROS, providing precise intracellular delivery of antioxidants into specific endothelial organelles involved in the ROS signaling and amelioration of specific proinflammatory vascular pathways.^[^ ^59^ ^]^ Targeting antioxidant enzymes and other agents to the abnormal endothelium using affinity ligands binding to CAMs has demonstrated obviously improved therapeutic effect in a number of animal models.^[^ ^56^, ^58^, ^59^, ^60^, ^61^, ^62^ ^]^ Thus, vascular immunotargeting is important and useful strategy for improved nanotheranostics. It is worth noting that nanocarriers engaging non‐internalizable surface determinants on the endothelial cells also induce effective endocytosis, which is via complex uncanonical pathways controlled by nanocarrier size, valency, elasticity, and hydrodynamic conditions.^[^ ^63^ ^]^

#### 4.1.2 Nutrient Transporters‐Mediated Increase in Vascular Endothelial Transcytosis

Nutrient transporters on the vascular endothelium are crucial to maintain the normal functions of tissues, especially for the more energy‐consuming disease tissues, for actively taking in nutrients, such as sugars, amino acids, etc. These nutrient transporters can be potentially served as access to increase nanotheranostic agents across endothelium.^[^ ^9^ ^]^

BBB that constitutes with high expression of tight junction proteins and low transcellular endocytosis of the cerebral microvessel endothelial cells is the primary challenge against nanotheranostic agents to reach brain.^[^ ^9^ ^]^ 1n 2013, Davis and co‐workers used peptides to recognize the transferrin receptor on brain capillary endothelial cells to increase the BBB‐crossing ability of nanocarrier. Unfortunately, its brain accumulation rate was less than 1.0% dose g^−1^‐brain.^[^ ^64^ ^]^ In 2016, Huang and co‐workers used overexpressed albumin‐binding proteins (e.g., secreted protein acidic and rich in cysteine (SPARC) and glycoprotein 60 (gp60)) on tumor vessel endothelium and glioma to explore the usage in brain‐targeting delivery (Figure 4c). The albumin nanoparticles, which self‐assembled form albumin and some hydrophobic drugs (i.e., paclitaxel and fenretinide), can implement to increasingly penetrate BBB via SPARC‐ and gp60‐mediated biomimetic endothelium transport, improving the efficacy of brain tumor therapy.^[^ ^65^ ^]^ Glucose transporter‐1 (GLUT1), as the main energy source glucose transporter in the brain, is expressed at a remarkably high level in brain capillary endothelial cells.^[^ ^66^ ^]^ Currently, several nanotheranostic agents with glucose as the ligand to target GLUT1 have been reported for brain disease theranostics, unfortunately most of them showed low transportation ability into brain. Based on this, in 2017, Kataoka and co‐workers proposed an innovative approach through glycemic control to increase glucosylated nanoparticles crossing BBB into the brain.^[^ ^66^ ^]^ First, self‐assembled nanoparticles with glucose ligands on the surface were synthesized. Specifically, the oppositely charged pairs of PEG‐PAsp and positively charged PEG‐P(Asp‐AP) block copolymers, were blended and self‐assembled into the nanocarriers (Figure 4d). Glucose with density from 0%, 10%, 25%, to 50% were covalently linked at the surface of the nanocarriers. The real‐time extravasation of the nanoparticles crossing BBB was assessed via measuring the fluorescence intensity and distribution. As shown in Figure 4e, the red signals (fluorescence) were exhibited only inside of the blood vessel at 10 min, which is before the administration of the glucose solution. Next, at 60 min (30 min after the administration of the glucose solution), the fluorescence had already distributed almost the brain parenchyma and then its intensity further enhanced until 90 min. These observations indicated that the BBB crossing of glycosylated nanocarriers are boosted via the GLUT1 transporter, exhibiting a positive relationship with the blood glucose concentration.

#### 4.1.3 Biomimetic Nanotheranostic Agents

Some biomimetic nanotheranostic agents closely mimic biology, such as viruses or cells, in both size and functions, which retains the theranostic ability and endows many new advantages. For example, virus‐biomimetic agents can load enzymes, nucleic acids, and DNA, which protects them from degradation. Additionally, some cell membrane‐biomimetic agents can overcome biological barriers to improve the targeting ability.^[^ ^67^ ^]^ Some nanotheranostic agents are encapsulated into the endogenous biomaterials to closely mimic biological process, which will increase vascular endothelial transcytosis to overcome vascular obstacles for improved nanotheranostic outcomes.^[^ ^68^ ^]^

**Figure 5:**
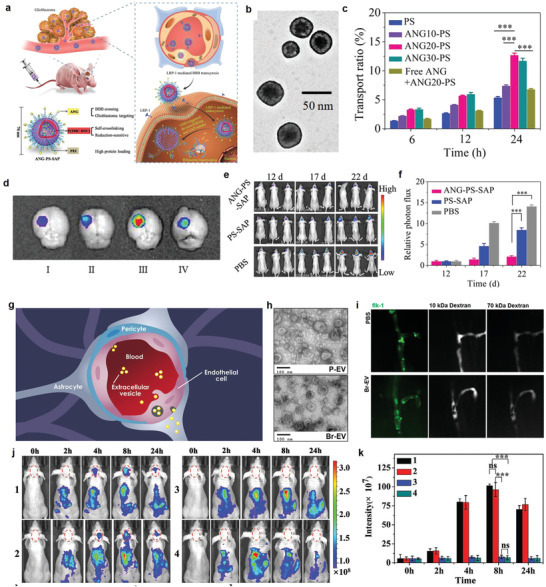
a) Illustration of components of ANG‐PS‐SAP and mechanism for in situ brain glioblastoma theranostic in mice. b) TEM images of ANG‐PS‐SAP. c) The transport ratios (%) of monolayer bEnd.3 cells for Cy5‐labeled PS, ANG10‐PS, ANG20‐PS, and ANG30‐PS at different incubation timeline. d) Brain fluorescence images confirmed that ANG effectively increased targeted ability of brain tumor ANG densities (I: 0%, II: 10%, III: 20%, IV: 30%). e) Bioluminescence images of mice with different treatments. f) Quantification of mice U‐87 MG‐Luc luminescence of (e). (a–f) Reproduced with permission.^[^ ^67^ ^]^ Copyright 2018, Wiley‐VCH. g) Illustration of transcytosis mechanism to cross intact BBB by using tumor‐derived extracellular vesicles. h) Electron microscopy images of P‐EV and Br‐EV). i) Fluorescent images of zebrafish brain vasculature. (g–i) Reproduced with permission.^[^ ^68^ ^]^ Copyright 2019, American Chemical Society. j) Real‐time fluorescence imaging of normal mice treated with (1) B16 cancer cell membrane‐coated indocyanine green‐loaded nanoparticle, (2) 4T1 cancer cell membrane‐coated indocyanine green‐loaded nanoparticle, (3) normal cell (COS‐7) membrane‐coated indocyanine green‐loaded nanoparticle, and (4) Indocyanine green‐loaded nanoparticle. k) Quantitative fluorescence intensity of the brain under different treatment at different time. (j,k) Reproduced with permission.^[^ ^69^ ^]^ Copyright 2020, Wiley‐VCH.

Overexpressed low‐density lipoprotein receptor‐related protein‐1 (LRP‐1) is observed in both brain capillary endothelial cells and glioblastoma cells, which is a particularly appealing target.^[^ ^67^ ^]^ Angiopep‐2 (ANG) peptide that derived from the Kunitz domain has a high affinity to LRP‐1. In 2018, Zhong and co‐workers fabricated the saporin (SAP)‐loaded ANG‐polymersomes (PS) nanoparticles (ANG‐PS‐SAP) as virus mimicking vesicles to high‐efficiency treat glioblastoma (**Figure** **5**a).^[^ ^67^ ^]^ ANG‐PS‐SAP exhibited a small size of 76 nm (Figure 5b). The PS are co‐self‐assembled from asymmetric triblock copolymer and diblock copolymer. PEG of the outer surface endows the good biocompatibility and long circulation of ANG‐PS. Optimal BBB penetration and glioblastoma‐targeting ability were achieved through ANG peptide on the surface of polymersomes via a longer PEG as linker. PEI of the inner side can efficiently encapsulate protein into the lumen of PS. Self‐crosslinked P(TMC‐DTC) in PS membrane was de‐crosslinked to release intracellular protein under cytoplasmic reductive condition. ANG‐PS is close with mimic viruses due to the following three characteristics. The first one is that its size is similar with viruses (Figure 5b). The second one is that a large amount of proteins and nucleic acids can be loaded into it, which also likes viruses to avoid them degradation (Figure 5c,d). The third is that ANG‐PS can home to the target site, internalize into target cells and release load for theranostics (Figure 5e,f). As desirable, ANG‐PS‐SAP can efficiently cross BBB to strongly inhibit glioblastoma growth without obvious side effects. Additionally, most cell types can release nanoscale extracellular vesicles (EVs). These EVs can closely mimic biological process to transfer a variety of theranostic agents to other cells, implementing improved theranostic outcomes. EVs have emerged as promising drug delivery vehicles for targeted therapy. Moses and co‐workers used native tumor‐derived EVs (isolating from parental and brain‐seeking MDA‐MB‐231 breast cancer cells, namely P‐EV and Br‐EV, respectively) to cross the intact BBB through the endothelial recycling endocytic pathway‐involved in transcellular transport (Figure 5g–i).^[^ ^68^ ^]^ EV‐driven transport provides the design guidelines of promising therapeutics vehicles for a variety of brain pathologies, such as brain malignancies, neurodegenerative disorders, and infectious diseases. In 2020, Liu and co‐workers used metastatic tumor cell membranes of brain as shell to fabricate the biomimetic nanocarriers (Figure 5j,k).^[^ ^69^ ^]^ The biomimetic core–shell structure can bind the receptors on brain endothelial cell surfaces, which effectively induces the nanoparticles to cross BBB through transcytosis mechanism, exhibiting high ability to transport nanotheranostic agents to cross the brain blood vessels for imaging and therapy of brain tumors.

#### 4.1.4 Electrostatic Adsorption Transcytosis

**Figure 6:**
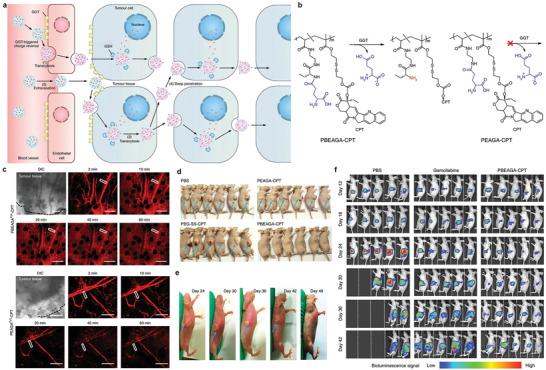
a) The sketches of the transendothelial and transcellular transport of the nanomedicine with cationization‐initiated transcytosis‐mediated active tumor penetration. b) The chemical structures of the GGT‐responsive PBEAGA‐CPT prodrug and the non‐GGT‐responsive PEAGA‐CPT prodrug. c) The extravasation and penetration of blood vessels of HepG2 tumors after the injection of PBEAGA^Cy5^‐CPT (red) (top) and PEAGA^Cy5^‐CPT (red) (down). d) Eradication effect of PBEAGA‐CPT for small‐sized tumors (≈100 mm^3^). e) Regression ability of PBEAGA‐CPT for large sized tumor (≈500 mm^3^). f) The survival of in situ pancreatic tumor‐bearing mice were significantly extended after treatment with PBEAGA‐CPT. (a–f) Reproduced with permission.^[^ ^73^ ^]^ Copyright 2019, Springer Nature.

Electrostatic adsorption transcytosis is initiated by electrostatic interaction between the plasma membrane of negative charge and cationic substances.^[^ ^48^, ^49^, ^50^, ^70^, ^71^, ^72^, ^73^, ^74^, ^75^ ^]^ In 2019, Shen  and co‐workers increased tumor penetration and treatment efficacy through the transcytosis of endothelial and cancer cells of nanomaterials (**Figure** **6**a).^[^ ^70^, ^73^ ^]^ Specifically, a *γ*‐glutamyl transpeptidase (GGT)‐responsive camptothecin–polymer conjugate nanotherapeutic agent PBEAGA‐CPT and the control (the non‐GGT‐responsive conjugate PEAGA‐CPT) were developed (Figure 6b). The overexpressed GGT on the surface of endothelial cell membrane can separate polymer conjugate nanoparticles to form cationic primary amines, which obtains a relatively homogeneous tumor distribution owing to caveolae‐mediated endocytosis and transcytosis for cationic (Figure 6c). The camptothecin–polymer conjugate can eradicate small solid tumors with the sizes of ≈100 mm^3^ (Figure 6d) and regress large tumors with clinically relevant sizes of ≈500 mm^3^ (Figure 6e). Compared with the commonly used chemotherapeutic drug gemcitabine, camptothecin–polymer conjugate obviously extended the survival of mice with in situ pancreatic tumor (Figure 6f), significantly enhancing therapeutic efficacy. Another work also demonstrated that enhanced transcytosis of a photodynamic therapeutic nanomedicine improves the therapeutic outcome. These studies thus suggests that transcytosis could be an important positive factor for designing nanotheranostic agents of cancer and other angiogenesis‐associated disease.^[^ ^71^ ^]^

#### 4.1.5 Decreased Flow Rates‐Induced Nanoparticle Uptake Increase into Endothelial Cells

Currently, most of standard in vitro experiment designs have no regard for the endothelial cells in the environment of in vivo dynamic bloodstream flow, so nanotheranostic agents are easy to settle onto the cells through gravity action, inducing uptake. However, the transport of these nanotheranostic agents in vivo highly depend on the blood flow rate, impacting their biodistribution.^[^ ^76^, ^77^, ^78^ ^]^ Mechanically responsive ability of endothelial cells of blood vessel may influence uptake ability of nanotheranostic agents under suffering different flow shear. The disorganized influence of shear leads to varying flow rates in diseased tissues, such as tumor vasculature. Thus, the traditional assays of flow shear lack results in overestimation theranostic effect in vivo.^[^ ^76^ ^]^

Chan and co‐workers adjusts interaction between nanoparticles and endothelial cells through using microfluidic system to change the flow rate of gold nanoparticles.^[^ ^76^ ^]^ Quantification of endothelial cell uptake for gold nanoparticles under different flow rates indicated that uptake of gold nanoparticles for human umbilical vein endothelial cells (HUVECs) decreased as increased flow shear. Endothelial‐cell‐binding ligand‐modified gold nanoparticles partially restore uptake degree to nonflow levels, demonstrating that increasing binding ability of gold nanoparticles and endothelial cells can weaken blood flow effects for uptake of gold nanoparticles.

### 4.2 Improved Nanotheranostic Agent Delivery to Disease Tissues by Bypassing Vascular Obstacles for Improved Nanotheranostic Outcomes

Since directly overcoming vascular obstacles are very difficult, and even if vascular obstacles are overcome, it may cause serious side‐effect, bypassing vascular obstacles to increase nanotheranostic agent delivery to disease tissues is another promise method to overcome vascular obstacles for improved nanotheranostic outcomes. Currently, the strategy of bypassing vascular obstacles is mainly applied in the delivery of biologics to central nervous system.^[^ ^79^ ^]^ Although the BBB is critical for maintaining the central nervous system homeostasis, it is also the main vascular obstacles for the nanothernostics of diseases in the central nervous system.^[^ ^80^ ^]^ Intranasal administration have demonstrated to bypass the BBB to noninvasively deliver therapeutic agents to central nervous system.^[^ ^81^ ^]^

**Figure 7:**
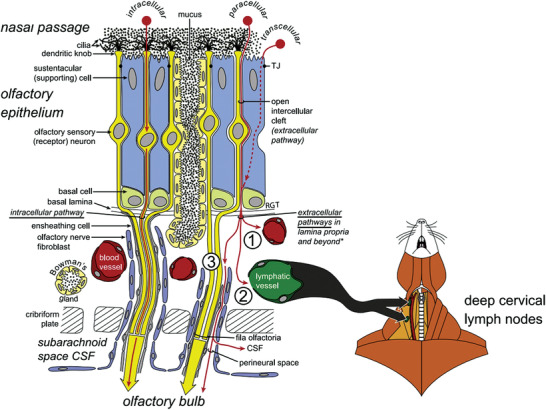
Possible pathways of drug delivery across the olfactory epithelium are shown in red by intranasal administration. Reproduced with permission.^[^ ^81^ ^]^ Copyright 2012, Elsevier.

**Figure** **7** showed the possible mechanism of intranasal administration to bypass BBB. Theranostic agents can be transported by paracellular or extracellular transport across the olfactory epithelial barrier to reach the lamina propria, where they are distributed by a variety of possible extracellular pathways.^[^ ^81^ ^]^

Glioblastoma is the most malignant primary brain tumor. In 2019, Paulmurugan and co‐workers fabricated a core–shell gold–iron oxide (as an MR imaging agent) nanoparticle, which was further functionalized with chitosan–cyclodextrin hybrid polymer, glioblastoma cell‐targeting T7 peptide (as a targeted group), and therapeutic miRNAs (antimiR‐21 and miR‐100).^[^ ^82^ ^]^ In vivo optical fluorescence and MR imaging demonstrated that such targeted theranostic system can bypass BBB to efficiently accumulate in brain through noninvasive intranasal targeted administration. The experiment of U87‐MG glioblastomas‐bearing mice also showed the targeted theranostic system obviously suppressed glioblastoma proliferation and prolonged survival rates of mice compared with the control group, or the nontargeted polyGIONsmiR‐100/antimiR‐21 group, or drug temozolomide chemotherapy alone.

### 4.3 Reducing Vascular to Nourish the Disease Tissues to Overcome Vascular Obstacle for Improved Nanotheranostic Outcomes

Blood vessels are a double‐edged sword for the treatment of diseases. Blood vessels not only delivery nanotheranostic agents into disease tissues, and provide accesses for patrolling immune cells, but also delivery nutrients and oxygen to the disease tissues to nourish their growth. So vascular nourishing disease tissue growth is also a huge obstacle for improved nanotheranostic outcomes of disease. Thus, scientists selectively embolize existing blood vessels and/or inhibit new vessel formation of disease tissues to reduce or eliminate vascular nutrient delivery to cancer and other angiogenic diseases tissues, which causes disease cells in a nutrient‐free state, eventually starving the disease cells to death. Such strategy is also used to combine with nanotheranostic agents to improve nanotheranostic outcomes.

#### 4.3.1 Selectively Embolizing Existing Blood Vessel of Disease Tissues

**Figure 8:**
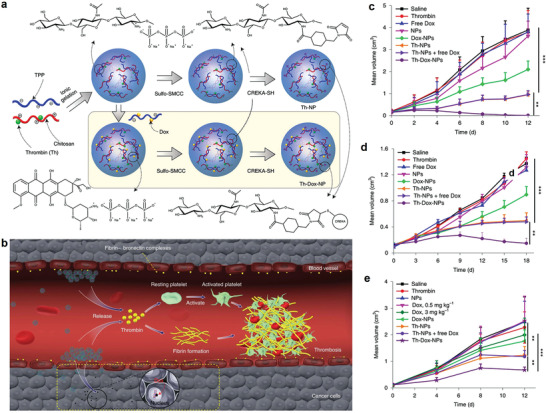
a) Design of combination therapy nanoparticles. b) Illustration of combination therapy mechanism of nanoparticles. The tumor‐growth curves of c) B16‐F10 melanomas, d) MDA‐MB‐231 human breast tumors, and e) MHCC97H human liver tumors in mice. (a–e) Reproduced with permission.^[^ ^27^ ^]^ Copyright 2020, Springer Nature.

Cancer vessels generally show similar properties. So targeting procoagulants‐induced selective thrombotic occlusion of vasculature in tumor tissues, a growing research field of cancer therapy, is a widely used strategy for many types of tumor therapy. In 2018, Zhao and co‐workers used DNA origami to construct an autonomous DNA nanorobot as nucleolin‐respond thrombin delivery systems.^[^ ^83^ ^]^ A DNA aptamer was functionalized on the surface of nanorobot to bind specifically expressed nucleolin protein on tumor‐associated endothelial cells. And the blood coagulation protease thrombin was loaded into the nanorobot cavity. At the tumor site, thrombin was released due to nucleolin‐opened the DNA nanorobot, which specifically activated tumor‐associated blood vessel coagulation and induced intravascular thrombosis, causing enhanced tumor necrosis and inhibition of tumor growth. However, drug‐induced vascular thrombosis in the tumor tissues cannot cause long‐term tumor eradication because tumor cells of the surviving tumor rim can still be nourished by adjacent non‐tumoral blood vessels and tissues, resulting in tumor regrowth. Thus, another work fabricated a chitosan‐based polymeric nanotheranostic agent (Th‐Dox‐NP) with a high capacity to perform a combination of tumor‐infarction therapy and chemotherapy.^[^ ^27^ ^]^ Th‐Dox‐NP was grafted a tumor‐homing pentapeptide (sequence: CREKA) to generate an active tumor tissue‐targeting ability (**Figure** **8**a). The fibrin–fibronectin complexes, uniquely overexpressed on the walls of tumor vessels and in tumor stroma, can specifically recognize CREKA peptide, which implemented thrombin‐controlled release at the tumor tissues form the nanotheranostic agents. It further initiated intratumoral thrombosis to reduce the nutrients supply of tumor, while a simultaneous accumulation of Dox also kills tumor cells, including the marginal tumor cells (Figure 8b). Compared with thrombin (Th‐NPs) or doxorubicin (Dox‐NPs) standalone, this combined chemotherapy and tumor‐infarction therapy (Th‐Dox‐NPs) obviously improved tumor growth suppression ability and reduced tumor recurrence (Figure 8c–e), improving the therapeutic index of coagulation‐based tumor therapy.

#### 4.3.2 Selectively Inhibiting New Vessel Formation of Disease Tissues

**Figure 9:**
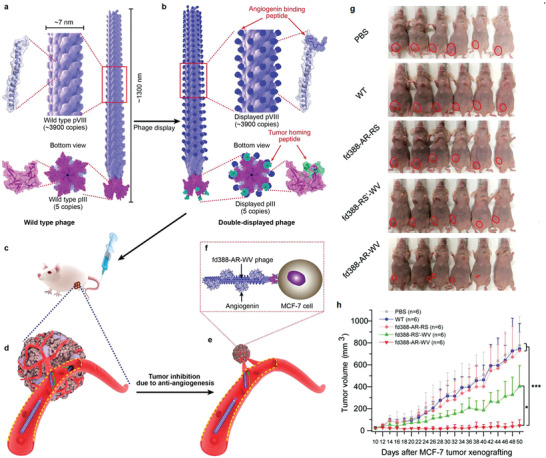
a–f) Illustration of using tumor‐homing angiogenin‐binding engineered phage nanofibers to selectively reduce new vessel formation of tumor tissues in the mouse model for tumor therapy. g) The images of the mice after 40th day treatment. h) The tumor volume changes in different treatment groups. Reproduced with permission.^[^ ^13^ ^]^ Copyright 2020, Wiley‐VCH.

Antiangiogenesis is a promising cancer treatment method, but it is limited due to the lack of tumor‐targeted ability of the current antiangiogenic drugs. In 2020, Mao and co‐workers used tumor‐homing angiogenin‐binding engineered phage nanofibers (fd388‐AR‐WV) to selectively reduce tumor angiogenesis, which implements targeted breast cancer therapy.^[^ ^13^ ^]^ Specifically speaking, the filamentous fd phage (termed fd388) (**Figure** **9**a,b) was first fabricated based on proteins and the core ssDNA. Furthermore, angiogenin binding peptide was displayed on the side surface of fd388 phages, the breast cancer‐homing peptides were modified at the tip of the fd388 phages. The phage nanofiber fd388‐AR‐WV can home to the breast tumor in situ and capture the angiogenin secreted by the tumor, thereby significantly preventing tumor angiogenesis (Figure 9c–f). The tumor size of the control groups (PBS, WT phage, fd388‐RS′‐WV phage, and fd388‐AR‐RS phage groups) were significantly bigger than that of the fd388‐AR‐WV‐treated group (Figure 9g,h), indicating that tumor‐homing angiogenin selectively inhibited new vessel formation of disease tissues.

### 4.4 Vascular Normalization to Overcome Vascular Obstacles for Improved Nanotheranostic Outcomes

The blood vessels of disease tissues, such as cancer, and rheumatoid and other diseases, are leaky and poorly methodic,^[^ ^15^, ^84^, ^85^ ^]^ which can enhance the interstitial fluid pressure of disease tissues to reduce blood supply, impairing the delivery of nanotheranostic agents.^[^ ^21^, ^86^ ^]^ Additionally, abnormal tumor vasculature causes a hypoxic tumor microenvironment (TME), which prevents the T effector cells to infiltrate into tumor and polarizes tumor‐associated macrophages to the immune‐inhibitory M2‐like phenotype, thus weakening the immune therapy ability of T effector cells.^[^ ^87^ ^]^ The approach of vascular normalization is calculated to remodel abnormal disease blood vessels to a more “normal” morphological state with recovered basal membrane and pericyte coverage, which make them less leaky and better organized, and decrease interstitial fluid pressure (**Table** **3**), improving the delivery and efficacy of theranostic agents.^[^ ^15^, ^20^, ^88^ ^,^ ^89^ ^]^

**Table 3:** Morphological and functional characteristics of the vasculature in normal tissue, without any agent‐treated tumor, during early stages of an antiangiogenic agent‐treated tumor tissues (normalized), and over long period of high doses of an antiangiogenic agent‐treated tumor tissues (regressing)^[^ ^21^ ^]^

Properties	Vessel type			
	Normal tissues	Tumor tissues (untreated)	Early stages of an antiangiogenic agent‐treated tumor tissues (normalized)	Over long period of high doses of an antiangiogenic agent‐treated tumor tissues (regressing)
Global organization	Normal	Abnormal	Normalized	Fragmented
Pericyte	Normal	Absent or detached	Closer to normal	Missing
Basement membrane	Normal	Absent or too thick	Closer to normal, some ghost	Ghost
Vessel diameter	Normal distribution	Dilated	Closer to normal	Closer to or less than normal
Vascular density	Normal, homogeneous distribution	Abnormal, heterogeneous distribution	Closer to normal	Extremely low
Permeability to large molecules	Normal	High	Intermediate	Variable
The contrast of microvascular pressure and microvascular pressure	Higher microvascular pressure and microvascular pressure	Approximately equal	Higher microvascular pressure and microvascular pressure	Low microvascular pressure
The contrast of plasma and interstitial oncotic pressure	Higher plasma oncotic pressure	Approximately equal	Higher plasma oncotic pressure	
pO_2_	Normal	Hypoxia	Reduced hypoxia	Hypoxia
Theranostic agent penetration	Uniform	Heterogeneous	More homogeneous	Inadequate

**Figure 10:**
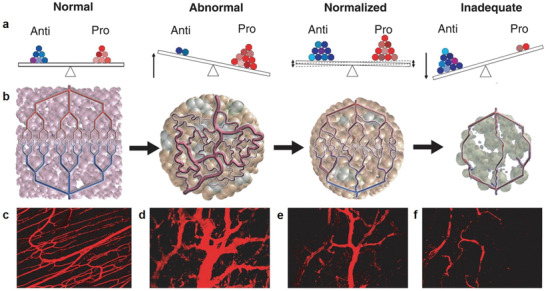
a) Balance state of proangiogenic and antiangiogenic factors in normal tissues, vasculature‐abnormal tumors, vasculature‐normalized tumors, and vasculature regressing tumor. b) The illustration of morphological characteristics of the vasculature in the four different tissues above. Fluorescence imaging of c) normal vasculature, and human colon carcinoma vasculature of mice at d) day 0, e) day 3, and f) day 5 after VEGR2‐specific antibody treatment. (a–f) Reproduced with permission.^[^ ^21^ ^]^ Copyright 2005, The American Association for the Advancement of Science.

The initial concept of vascular normalization was based pharmacological blockade studies of VEGF, while now its study has been extended to represent a homeostatic balance between the normal and abnormal blood vessel, as well as their respective microenvironmental and therapeutic sequelae. Clearly, this balance state is regulated by many important proangiogenic and antiangiogenic factors, but other biomolecules are also involved in a wide range of functions, including perivascular cell biology and the regulation of oxygen sensitivity (**Figure** **10**a).^[^ ^14^ ^]^ Such balance in pathological angiogenesis, for example, normalized tumor vasculature, also persists. Breaking this balance may cause vascular regression (Figure 10b–f).^[89]^ Additionally, the vasculature exhibits different morphological characteristics in normal tissue, without any agent‐treated tumor (abnormal), during early stages of an antiangiogenic agent‐treated tumor tissues (normalized), and over long period of high doses of an antiangiogenic agent‐treated tumor tissues (regressing). Thus, optimal time window for normalization therapy should be explored, so as to know the time window during which the vessels initially become normalized, and to understand how long they remain in that state. A study of VEGF receptor‐2 antibody‐treated human tumors growing in mice demonstrated such a “normalization window,” occurred transiently (about 6 days) to yield the best radiation therapy outcome.^[^ ^21^ ^]^ Additionally, the dose of antiangiogenic agents is also vital for the efficacy of vessel normalization therapy.^[^ ^21^ ^]^

#### 4.4.1 Inorganic Nanoparticles

**Figure 11:**
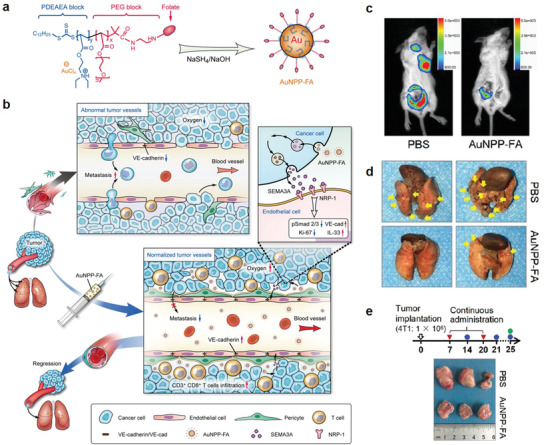
a) Components and synthetic route of AuNPP‐FA. b) Illustration of the mechanism of AuNPP‐FA‐induced tumor vasculature normalization and tumor metastasis decrease. c) In vivo imaging of 4T1 tumor cells at two weeks after PBS or AuNPP‐FA‐treated 4T1 tumor‐bearing mice, AuNPP‐FA‐treated tumor mice showed obviously decreased tumor sizes and metastases. d) Representative images of pulmonary metastatic nodules (yellow arrows) for PBS or AuNPP‐FA‐treated 4T1 tumor‐bearing mice. e) Representative tumor images from the PBS and AuNPP‐FA‐treated mice. (a–e) Reproduced with permission.^[^ ^19^ ^]^ Copyright 2020, American Chemical Society.

Some inorganic nanoparticles are frequently used as nanotheranostic agents, such as gold, mesoporous silica and titanium dioxide nanoparticles. However, some new biological effects of these nanoparticles are explored. In 2017, Setyawati and Leong used mesoporous silica nanoparticles to elegantly restrict the endothelial cells angiogenic behavior in a conventional drug‐free manner.^[^ ^18^ ^]^ Mesoporous silica nanoparticles as an antitumoral‐angiogenesis process showed a size‐dependent manner. Compared with the mesoporous silica nanoparticles of average primary sizes of 41 and 103 nm, group with an average size of 60 nm showed the best performance. Mesoporous silica nanoparticles induced intracellular reactive oxygen species level activates p53 signal, causing the inhibition of migration and invasion, proliferation, and survivability of the endothelial cells. Another work fabricated folic acid modified gold nanoparticles (AuNPP‐FA) (**Figure** **11**a),^[^ ^19^ ^]^ which can increase the level of semaphorin 3A (SEMA3A) of cancer cells, causing changes in some pathways of tumor vascular endothelial cells, such as, inhibition of Smad2/3 signaling, upregulation of VE‐cadherin and IL‐33, and downregulation of Ki‐67 (Figure 11b). These changes effectively induced tumor vessels normalization, which reduced vascular permeability, improved vascular perfusion, and relieved tissue hypoxia, significantly suppressing tumor metastasis (Figure 11c,d). Besides inhibiting the proliferation of tumor cells, AuNPP‐FA also enhanced immunotherapeutic response because of the improved infiltration of CD3+CD8+ T lymphocytes, suppressing the tumor growth (Figure 11e). Thus, AuNPP‐FA has opened a new door to future clinical therapy of cancer.

#### 4.4.2 Cytokines

**Figure 12:**
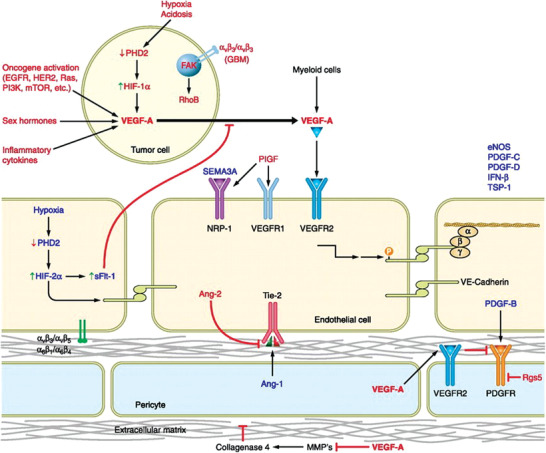
Various factors of tumor tissues that promote or inhibit the vascular normalization phenotype. The red is a cytokine that causes characteristic vessel abnormalities, and the blue is a cytokine that promotes the normalization phenotype. Reproduced with permission.^[^ ^14^ ^]^ Copyright 2011, American Physiological Society.

VEGF is the major angiogenic factor for vascular abnormalities, also known as VEGF‐A. Tumor vascular normalization are associated with a large number of cytokines (**Figure** **12**), mainly including two groups: one group is a cytokine that causes characteristic vessel abnormalities (red in Figure 12), including Angiopoietin (Ang); epidermal growth factor receptor (EGFR), focal adhesion kinase, glioblastoma multiforme, human epidermal growth factor receptor 2, hypoxia inducible transcription factor, phosphoinositide‐3‐kinase; placental growth factor, regulator of G protein signaling 5. The other group is a cytokine that promotes the normalization phenotype (blue in Figure 12), including endothelial nitric oxide synthase, interferon, platelet‐derived growth factor (PDGF), prolyl hydroxylase domain protein, SEMA, soluble VEGFR1, and thrombospondin.^[^ ^14^, ^90^, ^91^ ^]^

**Figure 13:**
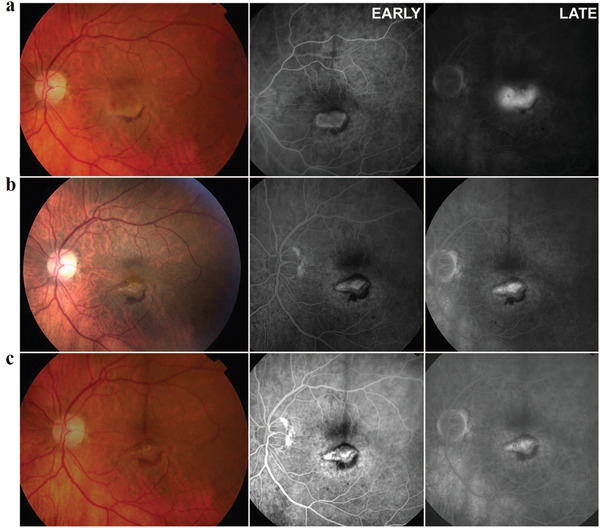
Early and late‐phase fluorescein images of the blood vessel of the eye. a) Baseline; bevacizumab injection given at this visit. b) Month 1 follow‐up visit; bevacizumab injection No. 2 given at this visit. c) Month 3 follow‐up visit, 2 months after the second bevacizumab injection; bevacizumab injection given at this visit. Reproduced with permission.^[^ ^93^ ^]^ Copyright 2006, Wolters Kluwer Health, Inc.

Jain and used VEGF receptor‐2 blocking antibody DC101 to repair the abnormal vessels of orthotopic mammary tumors to study nanoparticle penetration rates in vivo in 2012.^[^ ^92^ ^]^ The results demonstrated vascular normalization reduced the sizes of pores of vessel walls, which further lowered the interstitial fluid pressure in tumors, impeding the delivery of larger nanoparticles (≈125 nm) but improving the delivery of smaller nanoparticles (≈12 nm). This study indicated that vascular normalization will improve theranostic effect of smaller (≈12 nm) agents. Additionally, vasculature normalization is also effective for the treatment of other diseases, such as neovascular age‐related macular degeneration. Another work evaluated the efficacy of intravitreal bevacizumab (a full length, humanized monoclonal antibody against VEGF, also binds and inhibits all the biologically active forms of VEGF) for the treatment of neovascular age‐related macular degeneration (**Figure** **13**).^[^ ^93^ ^]^ Fluorescence imaging of blood vessel of the left eye exhibited an expanding and significantly classic neovascular lesion with obvious leakiness (Figure 13a). After one‐month treatment with intravitreal bevacizumab, fluorescein angiography image revealed a lesser neovascular lesion with significantly reduced leakage (Figure 13b). Two months after the second injection of bevacizumab, no leakage from the lesion was observed through fluorescein angiography (Figure 13c). These results demonstrated that antiangiogenic therapy may be a cost‐effective strategy for the treatment of many nonmalignant diseases.

#### 4.4.3 Nitric Oxide (NO)

NO in endothelial cells not only maintains endothelial function and vascular homeostasis, but also mediates angiogenesis. Thus, creating gradual change of perivascular NO can normalize disease vasculature, causing improved disease treatment.

**Figure 14:**
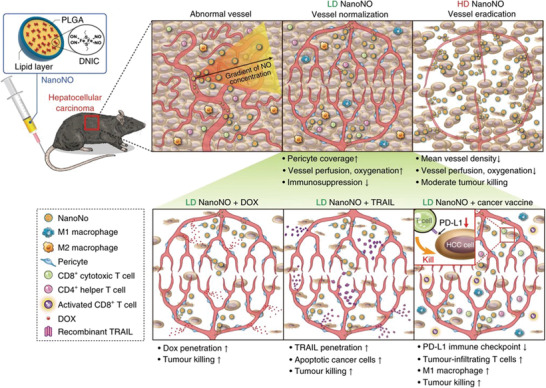
Scheme to show the mechanism that NanoNO suppresses hepatocellular carcinoma (HCC) progression in mice. LD, low dose; HD, high dose. Reproduced with permission.^[^ ^94^ ^]^ Copyright 2019, Springer Nature.

Chen and co‐workers fabricated a NO delivery system (NanoNO) that was component of glycol (PEG) polymer surface, and the biodegradable poly(lactic‐*co*‐glycolic acid) (PLGA) polymer and the NO donor dinitrosyl iron complex core (**Figure** **14**).^[^ ^94^ ^]^ Low‐dose NanoNO effectively normalized tumor vasculatures, which ameliorated immunosuppressive TME, suppressing metastasis and improving the cancer therapy efficacy of three modalities, including chemotherapy, a vaccine‐based cancer immunotherapy and tumor necrosis factor‐related apoptosis‐inducing ligand‐based biological therapies. NanoNO‐mediated angiogenesis has opened up a new door for various cancer therapy.

#### 4.4.4 Small Interfering RNAs

Small interfering RNAs (siRNA), a less than 30 nucleotides long mRNA sequence, is a kind of gene silencing method, providing new avenues to treat some gene‐associated angiogenesis diseases.^[^ ^95^ ^]^ It has been shown that siRNA can inhibit angiogenesis and cause vessels normalized, which is a new and effective strategy to cure solid tumors. Nie and co‐workers developed a nanoparticle for steady delivery of Nogo‐B receptor siRNA to normalize tumor vessel and suppress metastasis.^[^ ^96^ ^]^ Nogo‐B receptor is important for regulating vascular development, angiogenesis, and the epithelial‐mesenchymal transition of cancer cells. Silencing of Nogo‐B receptor effectively reverted the epithelial‐mesenchymal transition process of breast cancer cells through suppressing endothelial cell migration and tubule formation.

siRNA‐mediated vessel normalization presents new opportunities and requirements against some gene‐associated angiogenesis diseases, resulting in it toward a more efficient and successful clinical application in the future. The accompanying challenge is to develop desirable nanocarriers to protect siRNAs from degradation in circulatory system for high efficiency delivery into target cells.

### 4.5 Vascular Destruction to Overcome Vascular Obstacles for Improved Nanotheranostic Outcomes

Disrupting vasculature of disease tissues can increase the vascular permeability, which facilitates the nanotheranostic agents to cross the vascular obstacles. It increases the accumulation of nanotheranostic agents in target tissues to improve the theranostic outcomes. The blood vessels have been disrupted by various stimuli, such as physical forces, hyperthermia, radiation, ultrasound, or physiological agents.^[^ ^9^ ^]^

#### 4.5.1 Nanomaterial‐Induced Endothelial Leakiness

The vascular endothelium as a semipermeable barrier regulates tissue fluid homeostasis, leukocyte traffic and nutrients across the vessel wall.^[^ ^17^ ^]^ Nanomaterial‐induced endothelial leakiness (NanoEL) includes two different pathways: the first is transcellular pathway through caveolae‐mediated vesicular transport, and the second is paracellular pathway via interendothelial junctions. The transcellular pathway of NanoEL is dependent on nanomaterial‐induced oxidative stress or apoptosis. Such kind of nanomaterials, such as metal oxide,^[^ ^17^ ^]^ nanodiamond,^[^ ^97^ ^]^ and carbon nanotubes^[^ ^98^ ^]^ increase intracellular reactive oxygen species (ROS), which induces cytoskeletal damage or apoptosis. It further induces shrinkage and leakiness of endothelial cells to cause vascular destruction to regulate vascular obstacles, which allows more nanotheranostic agents to penetrate through the vascular barrier to reach the diseased tissues.^[^ ^17^, ^38^, ^97^, ^98^ ^]^

**Figure 15:**
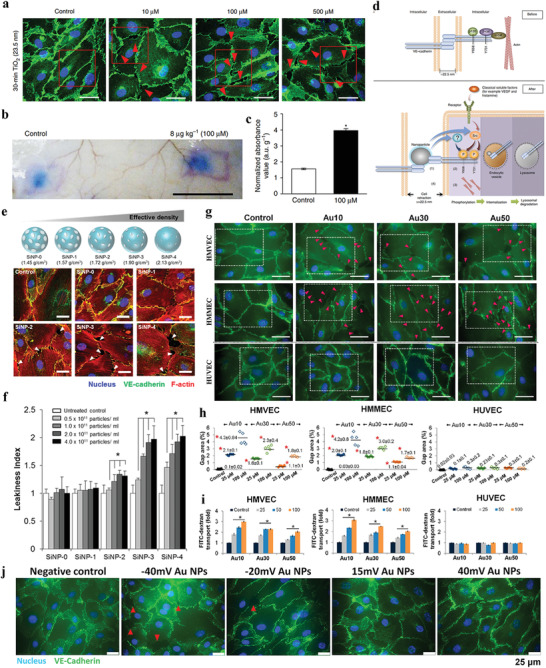
a) NanoEL effect of TiO_2_ nanoparticles under different dose for endothelial cells within 30 min. b) NanoEL effect of TiO_2_ nanoparticles in mice's skin blood vessel, vehicle buffer (Control), and TiO_2_ nanoparticles were subcutaneous injection. Evan's blue dye was tail vein injection. c) Quantification of Evan's blue extravasation in (b). d) Proposed mechanism of TiO_2_ NanoEL. (a–d) Reproduced with permission.^[^ ^99^ ^]^ Copyright 2013, Springer Nature. e) Fluorescence images of HMVECs were treated with different density but consistently sized SiNPs. f) Endothelial leakiness index under different SiNPs dose treatment. (e,f) Reproduced with permission.^[^ ^100^ ^]^ Copyright 2017, American Chemical Society. g) The different sized Au nanoparticles induced leakiness gaps for HMVECs, HMMECs, and HUVECs. h) NanoEL gaps were dependent on the Au nanoparticle size and the cell type. i) FITC‐dextran penetration increase is dependent to Au nanoparticle dose. (g–i) Reproduced with permission.^[^ ^45^ ^]^ Copyright 2017, American Chemical Society. j) Representative immunofluorescence images of leakiness gaps induced by Au nanoparticles (10 × 10^−6^ m, 15 min) with different charges (−40, −20, 15, and 40 mV). (j) Reproduced with permission.^[^ ^101^ ^]^ Copyright 2018, American Chemical Society.

In 2013, Leong and co‐workers proposed a new paracellular pathway mechanism about NanoEL, which is independent of cellular ROS.^[^ ^99^ ^]^ They found that TiO_2_ can induced leakiness of endothelial cells and blood vessel (**Figure** **15**a–c). In this mechanism, nanomaterials exerted a disruptive force to break the homophilic interactions of critical adherens junction proteins such as VE‐cadherin (Figure 15d). Detailly, the endothelial cell–cell adherens junction is maintained through stable VE‐cadherin before NanoEL. Ternary complex of *β*‐catenin, p120 and VE‐cadherin stabilizes the adherens junction. After NanoEL, TiO_2_ nanoparticles disrupt VE‐cadherin junction, inducing the phosphorylation of Y658 part of VE‐cadherin, while the Y731 residue is phosphorylated by Srckinase. The phosphorylation of two residues opens ternary complex interaction. The loss of interaction of the ternary complex destabilizes actin and causes actin remodeling, which results in cell retraction and leakiness (Figure 15d).

In 2017, Leong and co‐workers further demonstrated that an increased density but consistently sized silica nanoparticles (SiNPs) can directly increase the impinging force exerted into endothelial cell–cell adhesion (Figure 15e), which could directly increase the destruction of VE‐cadherin adherens junctions, achieving improved endothelial leakiness.^[^ ^100^ ^]^ The critical threshold of particle density was confirmed to be between 1.57 to 1.72 g cm^−3^ (Figure 15f). In 2017, Leong and co‐workers further investigated the endothelial leakiness effect of gold (Au) nanoparticles of different sizes, namely, Au_10_ (≈10 nm), Au_30_ (≈30 nm), and Au_50_ (≈50 nm) for Human mammary endothelial cells (HMMECs) and human skin endothelial cells (HMVECs).^[^ ^45^ ^]^ Under treatment with the same Au dose of 25 × 10−3 m for 1 h, the quantification analysis of the formed NanoEL gap area showed that HMMECs are more sensitive to generate more leakiness by Au nanoparticles than HMVECs (Figure 15g–i). Additionally, with the increased Au particle size, the endothelial leakiness gap area gradually decreases under the same dose of Au nanoparticles. In 2018, Leong and co‐workers used Au nanoparticles (≈24 nm) with different surface charges, including highly negative (−40 mV), low negative (−20 mV), low positive (+15 mV), and highly positive charge (+40 mV), to tune endothelial leakiness.^[^ ^101^ ^]^ The results showed that the Au nanoparticles with high negative charge surface induce significantly higher endothelial leakiness gap than Au nanoparticles with positive charge within 15 min (Figure 15j). These studies showed that vascular endothelial leakiness gap area is related to the mass density, size, and surface charge of nanoparticles, which helps us to use different nanoparticles to produce expected NanoEL effect to regulate vascular obstacles, implementing improved nanotheranostic outcome, making a provisional vasculature gap to improve paracellular movement of molecule across the vascular barrier.

Although NanoEL exhibited many advantages to regulate vascular obstacles for improved nanotheranostic outcome, we also need to examine its side‐effect. In 2019, Peng et al. administrated titanium dioxide, silica, and gold nanoparticles into mice by intravenous injection.^[^ ^102^ ^]^ Both intravasation and extravasation of cancer cells was obviously increased in vivo, which promoted the cancer metastasis. Thus, how to effectively utilize NanoEL to improve nanotheranostic outcome and avoid its side‐effect will require considerable thought in the future.

#### 4.5.2 Radiation

Radiation is the emission or transmission of energy in the form of waves or particles through space or through a material medium. Radiation‐increased nanotheranostic effect is mainly due to radiation‐caused changes in vasculature, cell signaling, and immune cells. Radiation treatment also caused the increased expression of VEGF, which enhances vascular leakiness. Radiation‐induced vascular endothelial cell apoptosis may also cause the increased vessel leakiness. These reasons also increase nanotheranostic agents crossing the vasculature to reach a large fraction of disease tissues, improving nanotheranostic effect. Additionally, radiation‐decreased interstitial fluid pressure induces transvascular pressure gradients, which is favorable for extravasation of nanotheranostic agents.

**Figure 16:**
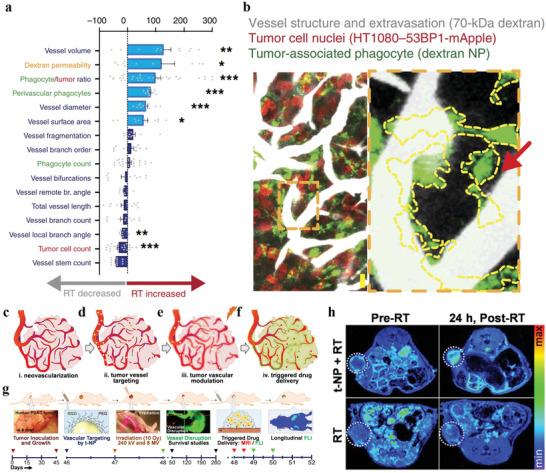
a) Image features of blood vessel with changed radiation. b) Vascular bursting (red arrows) via a cascade of changes after radiation. (a,b) Reproduced with permission.^[^ ^104^ ^]^ Copyright 2017, The American Association for the Advancement of Science. c–g) Scheme to show radiation‐induced tumor vascular change to increase drug delivery. h) T1‐weighted MR imaging demonstrated radiation therapy (RT) induced enhanced uptake of gold nanoparticles (t‐NP) than the RT‐only group. (c–h) Reproduced with permission.^[^ ^105^ ^]^ Copyright 2019, Springer Nature.

In 2004, Davies Cde et al. studied tumor growth and microdistribution of liposomal doxorubicin with/without radiation in orthotopic and subcutaneous human osteosarcoma xenograft. The results indicated that doxorubicin uptake (2–4‐fold), extravasation distance and deeper drug tumor penetration in radiation‐treated mice groups are all increased compared with those without radiation treatment. Radiation treatment also increased the number of apoptotic tumor cells and delayed tumor growth (**Figure** **16**a,b).^[^ ^103^, ^104^ ^]^ Another example was that radiation treatment resulted in a twofold higher uptake of both 70 and 120 nm iron oxide nanoparticles relative to non‐irradiated controls due to radiation‐caused lowered interstitial fluid pressure and improved vascular permeability.^[^ ^9^ ^]^ In 2017, Weissleder et al. demonstrated that a single low dose of radiation (2 Gy) enhanced vascular bursting via a cascade of changes to the tumor vasculature, increasing cytotoxic T‐lymphocyte membrane‐coated nanodrug carrying paclitaxel delivery (Figure 16c–g).^[^ ^105^ ^]^ It resulted in the significantly increased tumor therapy effect (tumor growth inhibition: ≈88.50%) than the only nanodrug‐treated group (tumor growth inhibition: 56.68%). In 2019, Berbeco and co‐workers used clinical radiation to activate the tumor endothelial‐targeted gold nanoparticles to selectively induce a tumor neovessel damage in a human pancreatic adenocarcinoma tumor model, which obviously increased tumor vascular permeability, causing more than twofold increase in nanodrug delivery (Figure 16h).^[^ ^105^ ^]^

Despite radiation have some advantages, radiation is difficult to operate without affecting the neighboring tissue, making it less favorable for the treatment of disease tissues. Thus, especially at higher doses (≥15 Gy), radiation carries risks including fibrosis, infertility, and bowel damage, should be given more considerations. Encouragingly, the lower dose (<5 Gy) was still effective in enhancing nanotheranostic agent delivery.^[^ ^104^ ^]^

#### 4.5.3 Ultrasound

Ultrasound (US) is a noninvasive and spatial‐temporal technique.^[^ ^106^ ^]^ US in combination with microbubbles can regulate vascular obstacles to improve nanotheranostic outcome, because it disrupts diseased vasculature via high temperatures, pressures expansion, compression of microbubbles burst, microstreams, and acoustic radiation forces. Vasculature disruption boosts vasculature leakiness, which increases the accumulation and extravasation distance of theranostic agents, improving theranostic outcome.^[^ ^107^, ^108^, ^109^ ^]^ Additionally, ultrasound without microbubbles also disrupts vasculature to some extent, which will also increase leakiness.

**Figure 17:**
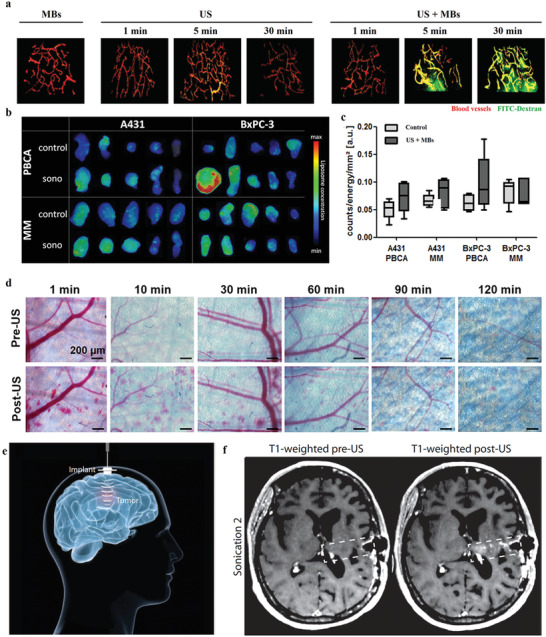
a) The effect of the vascular permeability for brain blood vessel with MBs treatment under ultrasonic activation upon 1 min, 5 and 30 min. Reproduced with permission.^[^ ^110^ ^]^ Copyright 2014, Wiley‐VCH. b) Sonoporation enhances liposome accumulation in A431 and BxPC‐3 tumors compared to control. c) Quantification of liposomes accumulating. (b,c) Reproduced with permission.^[^ ^111^ ^]^ Copyright 2016, Elsevier. d) The disruption of blood vessel over time under US‐stimulated MBs. Reproduced with permission.^[^ ^112^ ^]^ Copyright 2018, American Chemical Society. e) Scheme to show the US device. f) The T1‐weighted images are before and after sonication. (e,f) Reproduced with permission.^[^ ^113^ ^]^ Copyright 2016, The American Association for the Advancement of Science.

Lammers et al. investigated the vascular permeability effect of fluorescein isothiocyanate‐dextran (FITC‐Dextran, 70 kDa) nanoparticles using brain blood vessel as vascular model with poly(butyl cyanoacrylate)‐based microbubbles (MBs) treatment under ultrasonic activation. The results showed FITC‐Dextran (70 kDa) nanoparticles can efficiently pass across the blood vessels upon the combination of MBs with US for 5 and 30 min (**Figure** **17**a).^[^ ^110^ ^]^ Theek et al. found that US combined with MBs can increase vessel permeabilization to obviously improve the accumulation and penetration of nanoliposome in tumors with low EPR effect.^[^ ^111^ ^]^ Although the baseline variability in EPR of tumor may affect the increase of accumulation and penetration of nanoliposome, a trend toward positive effects was still observed. The amount of nanoliposomes treated by the combined of US and poly(butyl cyanoacrylate) (PBCA)‐based MBs in both A431 and the BxPC‐3 tumors increased up to 20–50% than the untreated tumors at 48 h after the i.v. injection of nanoliposomes (Figure 17b,c). Correspondingly, about more than 8% nanoliposomes enhancement in deep compartment (10–30 µm) of A431 tumors was observed after treatment with both PBCA‐ and MicroMarker (MM)‐based MBs under US. While enhancements of 4% and 12% in A431 tumor‐bearing mice was found in the deeper compartments (30–50 µm) for PBCA and MM with US, respectively. These results exhibited the enhanced ability of US and MBs for nanoliposomes to extravasate out of the blood vessels into the tumor interstitium, improving the accumulation and penetration of nanoliposomes. Ho et al. used focused US and lipid‐shell MBs to evaluate the efficiency of the antivascular effect via intravital imaging to explore the optimal US‐stimulated MB destruction parameters.^[^ ^112^ ^]^ The results showed that the US treatment intensity at 2 × 10^7^ microbubbles per mouse 7 MPa (3 cycles) and time duration of 60–120 min can cause the disruption of 21–44% of vessels smaller than 50 µm (Figure 17d). Such study based on quantitative investigations of US parameters and MBs dose regulation also indicated large vessels, especially in the tumor rim, still can be disrupted by regulating US‐stimulated MB destruction, which might reduce the probability of tumor recurrence. Carpentier et al. used an implantable US device system (Figure 17e), SonoCloud, to perform an US dose‐escalating phase 1/2a clinical interim trial before treatment with carboplatin for recurrent glioblastoma patients.^[^ ^113^ ^]^ The results showed that pulsed US in combination with MBs disrupted the cerebral microvessels to open the BBB of each patient (Figure 17f). This method can repeatedly open BBB. Additionally, such method has no obvious acute hemorrhage, ischemia, or edema symptom in immediate postsonication susceptibility‐weighted angiography, diffusion, or fluid‐attenuated inversion recovery sequences, demonstrating its safe and well tolerated in patients with recurrent glioblastoma. Thus, this method holds great promise to optimize nanotheranostic agent delivery in the brain.

Despite US exhibited many advantages for biomedical applications, its side‐effect also should be noticed.^[^ ^114^ ^]^ For example, excessive exposure to US at certain levels and frequencies may induce inflammatory response and cellular tissue damage.^[^ ^114^ ^]^

#### 4.5.4 Magnetic Forces

Magnetic field enables better practicability in deep biological tissues due to no attenuation in biological tissues. Additionally, the intensity of magnetic field is easy to control. A focused magnetic field gradient can be generated at any location in the body. So magnetic nanoparticles have been widely used in clinical practice.

**Figure 18:**
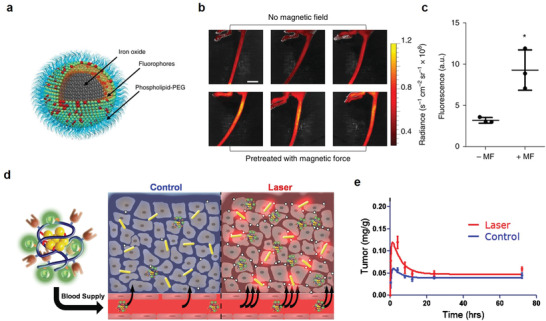
a) Scheme to show the structure of magnetic nanoparticles (MNPs). b) The accumulation of ICG without and with the magnetic force treatment. c) Quantification of ICG accumulation through quantifying fluorescence intensity in (c). (a–c) Reproduced with permission.^[^ ^115^ ^]^ Copyright 2017, Springer Nature. d) Scheme to show the photothermal effect to effectively increases the pore size in the tumor vasculature for increased tumor accumulation and retention. e) Photothermal effect causes increased vascular pore size to enhance burst accumulation occurred of nanotheranostic agents in tumor. (d–e) Reproduced with permission.^[^ ^117^ ^]^ Copyright 2012, Elsevier.

In 2017, Qiu et al. investigated the changes in structure and function of the vascular endothelial barrier under the treatment of intracellular magnetic force with a mouse lateral tail vein model.^[^ ^115^ ^]^ The intracellular iron oxide nanoparticle‐generated intracellular magnetic forces under an external magnetic field can disrupt endothelial adherens junctions and reorganize F‐actin fibers (**Figure** **18**a), which increased vascular endothelial leakiness, improving the paracellular transport pathway (Figure 18b,c). The magnetic control method shows the great potential to deliver therapeutic agents to specific biological tissues in our body for improved disease interventions.

#### 4.5.5 Light

In vivo application of light exhibits unique advantages, such as low cost, simple operation, noninvasive, excellent spatiotemporal resolution, and easy control.

In 2014, Zhen et al. developed a new strategy‐based photodynamic therapy (PDT) to selectively enhance leakiness of tumor vessel to boost tumor therapy outcome.^[^ ^116^ ^]^ ZnF_16_Pc‐loaded arginylglycylaspartic acid (RGD)‐modified ferritin (P‐RFRTs) can specifically target to the tumor endothelium to release ^1^O_2_. After 637 nm light irradiance, tumor xenograft models showed improved nanoparticle accumulation in tumor without obvious side‐effects. Additionally, scanning electron microscopy images also showed the irradiated tumor showed more large fenestrae on the blood vessel walls compared to the unirradiated groups. Thus, P‐RFRT‐mediated PDT demonstrated a new and safe approach to increase the EPR effect to improve therapy outcome.

Additionally, photoenergy can be transformed into photothermal energy to disrupt tumor blood vessels to increase vascular permeability.^[^ ^117^ ^]^ Gormley et al. used PEGylated gold nanorods as photothermal agents to disaggregate the cytoskeleton of endothelial cells (Figure 18d), which effectively increased the tumor microvascular fenestrae and the expression of heat shock proteins on cell surfaces. It resulted in increased tumor accumulation (Figure 18e) and retention of nanotheranostic agents, which improved photothermal therapy outcomes.^[^ ^118^ ^]^

#### 4.5.6 Temperature

As early as in 1985, Lefor et al. investigated the relationship between hyperthermia and vascular permeability effects of Walker carcinosarcomas and host liver tissue by quantifying optical density of Evans blue per gram tissues.^[^ ^119^ ^]^ Tumor vascular permeability had no obvious change compared with control levels under treatment of a nontherapeutic level at 40 °C. However, within the therapeutic range of hyperthermia at 43 °C, tumor vascular permeability significantly rose. Tumor permeability was increased with the increase of time under hyperthermia at 43 °C. The phenomenon was similar with changes from freeze‐thaw‐caused physical damage. Another study investigated the effects of the temperatures range (34–42 °C) and hyperthermia treatment scheduling (time between hyperthermia and drug administration as well as between consecutive hyperthermia treatments) for the extravasation of 100 nm nanoliposomes from tumor microvasculature in SKOV‐3 ovarian carcinoma‐bearing nude mouse.^[^ ^120^ ^]^ From 34 to 39 °C, the extravasation of were not observed form the tumor interstitium. From 40 to 42 °C, the extravasation of nanoliposomes was proportional to temperatures. While temperature was higher than 42 °C, resulting in vascular hemorrhage and stasis in tumor. After heating, nanoparticle extravasation was first increased and then decay back to baseline level at 6 h postheating. However, the nanoparticle extravasation remained almost same under the treatment with reheating at 8 h after an initial heating (42 °C for 1 h), probably due to the development of vascular thermotolerance. The results provided a guideline to increase vascular permeability for improving theranostic outcome by scheduling of hyperthermia. Additionally, microscopy images showed hyperthermia treatment also increased liposome extravasation distance from 3.7 ± 1.9  to 21 ± 2.5 mm, the therapeutic efficacy increased twofold. These results indicated that temperature is an effective method to improve nanotheranostic outcome.

#### 4.5.7 Electroporation

If blood vessel was exposed to electroporation (EP), the vascular diameter would be immediately reduced resulting in blood flow abrogation, followed by an increase in vascular permeability.

In 2018, Markelc et al. demonstrated that EP transiently reduced the cell–cell junction protein VE‐cadherin of monolayered murine endothelial cells bEnd.3. Within 20 min after EP, the quantity of VE‐cadherin has recovered to control levels.^[^ ^121^ ^]^ Under the treatment of clinically validated EP parameters, fluorescently labeled antibodies against PECAM‐1 (CD31) were further used to visualize endothelial cell–cell junctions with a dorsal window chamber model in C57Bl/6 mice. After EP treatment, the blood vessels started to constrict, which decreased the volume of labeled cell–cell junctions, causing increased vascular permeability for 70 kDa fluorescein isothiocyanate labeled dextran. Thus, EP treatment holds great promise for a facilitated vascular penetration of theranostic agents.

Furthermore, this event alone could possibly trigger the platelet activation cascade and formation of clots, leading to local inflammation.

#### 4.5.8 Extravasating Leukocyte

In 2019, Naumenko et al. investigated the extravasation patterns of fluorescent liposomes in tumor and normal skin.^[^ ^122^ ^]^ As it turned out, extravasated neutrophils can open vascular barrier, which obviously improves liposomes delivery to tumors. Microleakage was defined as a local perivascular nanoparticle deposition, which was observed both in tumor and normal tissues. Its formation usually happened within several minutes and remained nearly constant over time. Additionally, the microleakage‐caused extravasation type was limited to a specific area and spread within 20 µm distance from the vessel walls. Liposome fluorescence of microleakage area was evenly distributed and higher than the vessel lumen. However, the microleakage‐caused drug extravasation is useless for drug delivery to tumor tissues, but also may increase the risk of skin toxicity. By contrast, microleakage‐caused extravasation was a bigger interstitial area and not stable in time and space. Macroleakage‐enabled nanodrug delivery to spread deeper tissues about hundreds of micrometers from the vessel walls and mainly localized on the tumor–host interface. Neutrophils extravasation initiated both micro‐ and macroleakages. The analysis of 24 macro‐ and 320 microleakages of all studied tumor models suggested that the probability distribution of macroleakage was threefold higher within 50 µm of neutrophils. Compared with the vasculature near tumor cells, the 500–1500 µm vessels of the tumor edge showed increased microleakage counts. The peritumoral vessels 1000–1500 µm aside from the core had a profound peak of macroleakage counts. Thus, extravasation rate of peritumoral vessels was higher than that of intratumoral vessels. Liposome extravasation was more common in peritumoral blood vessels and associated with neutrophils infiltration. Neutrophil depletion caused decreased liposomes accumulation in tumors.^[^ ^123^, ^124^, ^125^, ^126^ ^]^

#### 4.5.9 Inducers of Angiogenesis

Numerous inducers of angiogenesis have been identified, including members of the fibroblast growth factor family, vascular permeability factor/VEGF, angiogenin, transforming growth factor alpha and beta (TGF‐*α* and ‐*β*), PDGF, tumor necrosis factor alpha, interleukins, chemokines, and angiopoietins. Angiogenesis has been observed in a wide variety of diseases, which include all the major causes of mortality in the West‐heart disease, cancer, stroke, vascular disease, and diabetes. Commonly, angiogenesis is regulated by vascular growth factors.^[^ ^127^ ^]^ VEGFs also directly stimulate increased vascular permeability to water and large molecular weight proteins and vasodilatation.^[^ ^128^, ^129^ ^]^

In 2012, Hsieh and co‐workers incidentally observed that intramyocardial injection of VEGFs allowed increased accumulation of systemically injected polystyrene nanoparticles in brain.^[^ ^130^ ^]^ In 2019, Hsieh and co‐workers further used a low dose of systemically injected recombinant human VEGFs to induce a short period of increased the BBB permeability, and restored into normal level within 4 h.^[^ ^131^ ^]^ Short period of increased BBB permeability increased the delivery of nanoparticles in various sizes to brain. Additionally, this strategy demonstrated that it can increase liposomal doxorubicin for improve therapy of glioblastoma multiforme with no noticeable systemic toxicity. Moreover, Iwasaki and co‐workers also demonstrated that VEGF‐C had the potential to drive meningeal lymphatic drainage, which enabled immune surveillance and T‐cell‐mediated immunity against brain tumors.^[^ ^132^ ^]^

#### 4.5.10 Mimicking Vascular Inflammation

Vascular inflammation is a common, complex mechanism involved in the pathogenesis of a plethora of disease conditions, including ischemia–reperfusion, atherosclerosis, restenosis, and stroke.^[^ ^133^ ^]^ Vascular inflammation represents a cascade of events including the endothelial expression of molecular determinants involved in the recruitment of leukocytes, which propagates the inflammatory process and increases vascular permeability. Inflammatory mediators such as TNF*α*, prostaglandin analogs, histamine or PAF, capable of enhancing vascular permeability, have been utilized to increase nanomedicine accumulation in tumors up to 2–6‐fold higher than that of the control group.^[^ ^134^, ^135^, ^136^, ^137^, ^138^ ^]^

**Figure 19:**
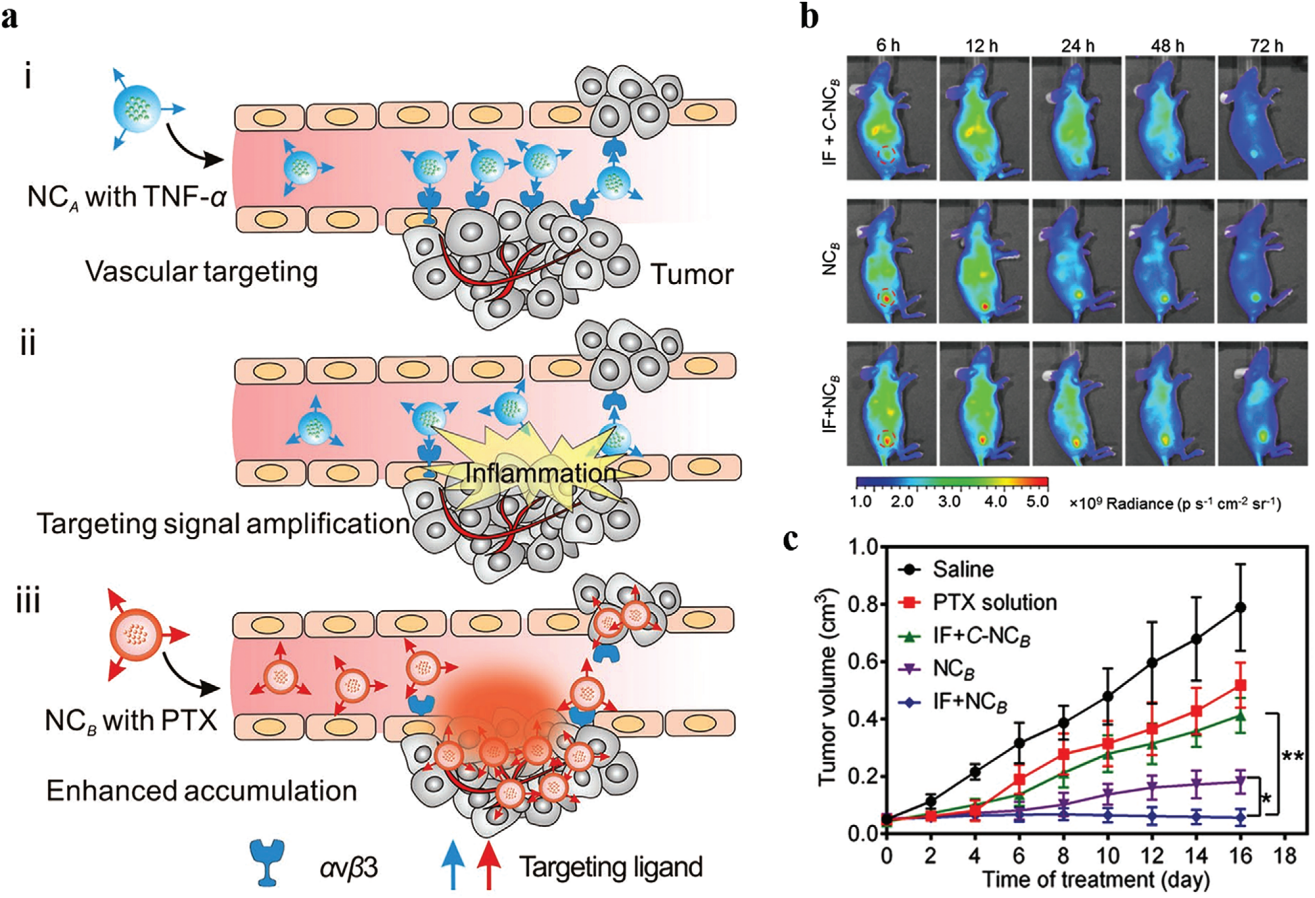
a) The illustration of the “relay delivery” strategy for amplifying targeting signal and increasing anticancer efficacy. b) Fluorescence imaging of the tumor bearing mice at 6, 12, 24, 48, and 72 h after tail vein injection of inflammation inducer NC_A_ (IF), Cy5.5‐labeled C‐NC_B_, and Cy5.5‐labeled NC_B_. c) The tumor growth curves after different treatment. (a–c) Reproduced with permission.^[^ ^139^ ^]^ Copyright 2017, Wiley‐VCH.

In 2017, Gu and co‐workers demonstrated a target‐enhanced theranostic strategy by a relay drug delivery.^[^ ^139^ ^]^ In this strategy, one module, the signal transmission nanocarrier A (named NC_A_), first can selectively bind integrins through the RGD to target to the tumor vasculature (**Figure** **19**a). Subsequently, the NC_A_‐encapsulated TNF‐*α* was released, which caused serious inflammation of endothelial cells of tumor vascular, further inducing the blood vessel damage. Another sequential module, an execution biomimetic nanocarrier B (named NC_B_), can respond the NC_A_‐broadcasted the targeting inflammation signal of tumor vascular endothelium, which reported the tumor location (Figure 19b). Then a large amount of NC_B_ accumulated into the tumor tissues. Under the acidic microenvironment of tumor, anticancer drug PTX was released from NCB to inhibit the functions of microtubules for enhancing anticancer efficacy (Figure 19c). The “relay delivery” strategy amplified targeting signal and enhanced anticancer efficacy.

#### 4.5.11 Platelet Depletion

In cancer tissues, the activity and numbers of platelets are enhanced, which simultaneously promotes tumor progression and metastasis. Additionally, overexpressed tissue factor in the TME can activate platelets to release proangiogenic factors, which activates the growth and propagation of endothelial cells of tumor blood vessel.^[^ ^140^, ^141^, ^142^ ^]^ Thus, platelets play a crucial role to maintain the integrity of tumor blood vessels, preventing tumor bleeding, and filtration and penetration of immune effector cells through controlling vascular tone and permeability.

**Figure 20:**
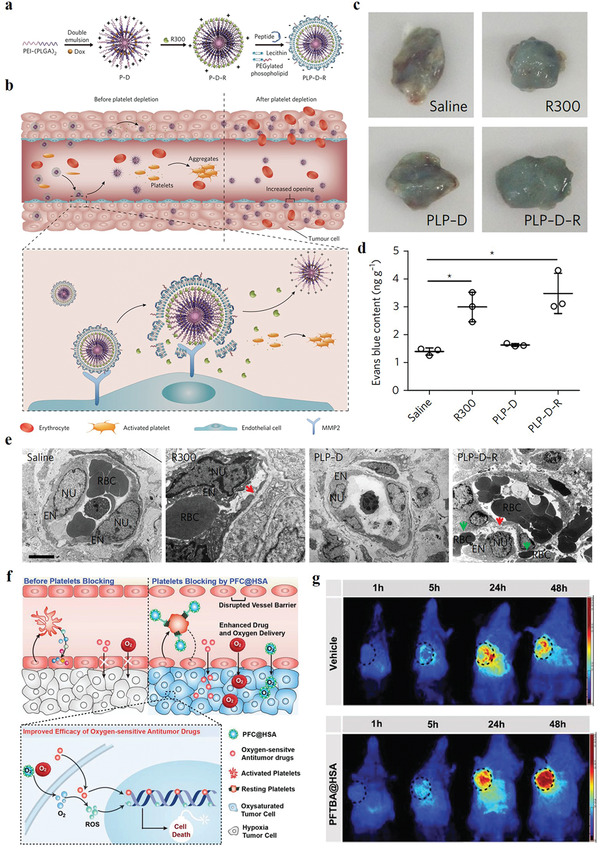
a) The illustration of the structure of MMP2‐cleavable PLP‐D‐R nanoparticles. b) The interaction mechanism between the PLP‐D‐R, and tumor endothelial cells. c) Tumor permeability study through intravenous Evans blue under different treatment. d) Quantization of Evans blue content of the tumors in (c). e) High‐resolution transmission electron microscopy images of blood vessels in MCF7 tumors 24 h after different treatment. (a–e) Reproduced with permission.^[^ ^143^ ^]^ Copyright 2017, Springer Nature. f) Scheme to show the mechanism of PFTBA@HSA‐mediated platelet blocking for improved therapy of oxygen‐sensitive antitumor drugs. g) Fluorescence imaging of mice with different treatment to demonstrate improved drug delivery by platelet blocking. (f,g) Reproduced with permission.^[^ ^144^ ^]^ Copyright 2017, Wiley‐VCH.

Platelets enhance the numbers of endothelial cells, reinforce the tight junction of endothelial cells, and optimize the arrangements of endothelial cells, causing damaged drug delivery in tumor surroundings. In 2017, Nie and co‐workers used a polymer–lipid–peptide‐based drug delivery system (PLP‐D‐R) to deplete local tumor‐associated platelets for improved tumor therapy and metastasis inhibition.^[^ ^143^ ^]^ An antiplatelet antibody R300 and the antitumor drug Dox were loaded into the biocompatible and biodegradable block copolymer poly(etherimide)–poly(lactic‐*co*‐glycolic acid)_2_ (PEI‐(PLGA)_2_)‐self‐assembled nanoparticles. Then lecithins, matrix metalloproteinase 2 (MMP2)‐cleavable peptides, and PEGylated phospholipids were further loaded as a shell layer (**Figure** **20**a). In the tumor tissues, MMP2, which overexpressed vascular endothelia and stroma of tumor, cleaved lipid–peptide shell to release R300 and Dox (Figure 20b). The R300 first induced microaggregation of 3–5 microaggregations, then platelet microaggregations can rapidly deplete by phagocytosis without causing the coagulation system activation, which facilitates vascular breaches, enhancing tumor permeability. Tumor permeability study of blood vessels in MCF7 tumors through intravenous Evans blue indicated that PLP‐D‐R increased Evans blue accumulation in tumor (Figure 20c,d). High‐resolution transmission electron microscopy images demonstrated that PLP‐D‐R‐treated vascular endothelia in tumors showed obviously compromised and red blood cell extravasation (Figure 20e). The results in vivo antitumor experiment also showed that this strategy leaded to improved tumor therapy and metastasis inhibition in mice. In conclusion, platelet depletion is an effective approach to improve nanotheranostic outcome.

#### 4.5.12 Other Approach to Destruct Blood Vessel

Besides the above methods, other methods initiating the above pathways could also enhance the vascular permeability to increase access of nanotheranostic agents to disease tissues for improved nanotheranostic outcomes.

Wu and co‐workers used human serum albumin (HSA) as perfluorotributylamine (PFTBA, one of perfluorocarbons (PFCs)) carrier to construct perfluorotributylamine‐based albumin nanoparticles (PFTBA@HSA) (Figure 20f).^[^ ^144^ ^]^ PFTBA may not only simultaneously increase drug delivery, but also relieve tumor hypoxia via effective platelet inhibition to disrupt tumor vessel barriers (Figure 20g), improving the antitumor efficacy of oxygen‐sensitive drugs.

### 4.6 Other Strategy to Overcome Vascular Obstacles for Improved Nanotheranostic Outcomes

The short blood circulation time is insufficient to get enough nanotheranostic agents to cross vasculature wall to disease cells due to the vascular obstacles. The clearance of liver sinusoidal endothelial cell of nanotheranostic agents from the blood circulation is the most important reason for short blood circulation time of nanotheranostic agents. Any strategy, either modulation of nanotheranostic agents or regulation of liver sinusoidal endothelial cell clearance ability, that prolongs blood circulation time can increase delivery efficiency to disease cells. Previous studies usually focused on modulating the surface of nanotheranostic agents. For example, the stealth coating of nanotheranostic agents, such as PEG‐coated agents, can increase blood circulation time to cross vasculature wall to reach disease cells, improving theranostic outcomes. Recently, a new strategy has been developed based on regulation of liver sinusoidal clearance ability, such as beforehand injecting scavenger ligands (fucoidan, polyinosinic acid (poly‐I), and dextran sulfate, agents used for receptor) to saturate the availability of clearance pathways. However, liver sinusoid has diverse clearance pathways, decorating a ligand on the nanotheranostic agents is difficult to saturate all clearance pathways.

In 2020, Kataoka and co‐workers used precisely designed oligo(l‐lysine) (OligoLys) conjugated with two‐armed PEG at its carboxyl end (two‐arm‐PEG‐OligoLys) for anchoring PEG to liver sinusoidal walls, which implement transient and selective stealth coating of liver sinusoidal endothelium (uncoated the endothelium of other tissues).^[^ ^145^ ^]^ PEG coating of liver sinusoidal endothelium effectively and simultaneously inhibited of various clearance pathways. Additionally, OligoLys with a two‐armed PEG was finally cleared from sinusoidal walls to the bile, while OligoLys with linear PEG was difficult to be cleared from the sinusoidal walls, possibly resulting in persistent disturbance of liver functions. Such transient and selective stealth coating of liver sinusoids with two‐arm‐PEG‐OligoLys effectively reduced clearance of nonviral and viral gene vectors by the liver sinusoids, thus improving agent efficiency in the target tissues.

## 5 Summary and Outlook

In this review, we comprehensively and timely summarize the current five general strategies and mechanisms about overcoming vascular obstacles for improved nanotheranostic outcomes. We also outline the paradigms for distinct strategies to overcome vascular obstacles. Although current strategies of overcoming vascular obstacles are powerful for improved nanotheranostic outcomes, they still suffer from some major challenges. For example, the strategy of increasing endothelium leakiness improves theranostic agent delivery to disease tissues, excessive endothelium leakiness also increases the possibility of malposition of nutrients, functional biomolecules, and cells on either side of the vessel wall. The escape of tumor cells via vessel wall to blood will cause tumor metastasis. The crossing of leukocytes in blood through vessel wall to tissues will cause autoimmune disease and corresponding complications. Thus, we need to balance the side‐effects as well as improve the theranostic outcomes. More importantly, new theories and related works for increasing endothelium leakiness are popping up during the last two years. For example, Chan and co‐workers demonstrated that up to 97% of nanoparticles could accumulate into tumors via an active endothelium transcytosis.^[^ ^5^ ^]^ Therefore, they think the relative contribution of native gaps of tumor for nanoparticles is unpredictable and variable. In the future, more studies should be done to demonstrate the detailed and clear mechanism for bringing forward some targeted strategies. Additionally, the toxicity (either potential or real) of frequently used materials of overcoming vascular obstacles remains the major roadblock to clinical translations. Although many scientists claim that their materials have no obvious toxicity, these studies are limited to short‐term toxicity test. The long‐term toxicity of these materials is still unknown. Developing clinically approved theranostic agents or endogenic materials in our body to overcome vascular obstacles may solve the concern of long‐term safety.

Bypassing vascular obstacles is a strategy with high efficiency. However, such strategy is mainly applied in the delivery of theranostic agents to central nervous system, limiting its application range.

Different with traditional passive delivery through free diffusion of nanotheranostic agents, increasing vascular endothelial transcytosis relies on an adenosine triphosphate‐mediated active endothelial transport process, which overcomes the limitation of passive delivery. For example, high interstitial fluid pressure in a solid tumor prevents free diffusion of nanotheranostic agents. Additionally, active vascular endothelial transcytosis may increase penetration depth and accumulation of nanotheranostic agents, providing a new opportunity to overcome vascular barrier for improved theranostics.^[^ ^48^ ^]^ Although promising, the efficiency of transcytosis is determined by the endocytosis‐mediated transcytosis rather than degradation pathway. Thus, more methods should be found to improve endocytosis‐mediated transcytosis.^[^ ^22^, ^48^ ^]^ Furthermore, plenty of vasculature‐targeted theranostic agents based on receptor‐ligand recognition and vascular pathological parameter activation have been developed to overcome the vascular obstacles. However, the overexpressed receptors or parameters in vascular disease tissues also exist in normal vascular tissues. Once injected, these targeted theranostic agents will be distributed throughout the whole body. The ideal situation is that the nonspecifically distributed theranostic agents never interact with normal blood vessel. In fact, most of current vasculature‐targeted theranostic agents are easy off‐target, which may cause bleeding, clots in the arteries, hypertension, uncommon thrombus, and gastrointestinal perforations. Thus, developing strategies of overcoming vascular obstacle with spatiotemporal control is crucial for clinical applications.

Selectively reducing or eliminating nutrition delivery to the disease tissues via vasculature is promising to improve the theranostic outcomes of nanomedicines, which prevents cancers from becoming more malignant and metastatic, and to increase the responsiveness to chemotherapy, immunotherapy, and radiation therapy. For patients who exhibit a poor response to chemotherapy or have recurrent or unresectable cancer and other angiogenic diseases. Unfortunately, this strategy needs to combine with other theranostic model to eradicated disease completely.

An important challenge of vascular normalization for clinical application is the optimization of dose and schedule for combination therapy for individual patients. Vessel normalization occurs transiently during a normalization window, general 6 days. Owing to narrow normalization window, it needs to carefully control the dosage and dosing period of antiangiogenic drug to adjust angiogenic and antiangiogenic balance for the appearance of vessel normalization. Thus, the strategy of vessel normalization is still challenging in clinic.

All the strategies mentioned above have been demonstrated with specific pros and contras for utility in specific conditions. The extent of specificity, safety, effectiveness, clinical utility, and mechanistic control about these strategies to overcome the vascular barriers are highly dependent on the nanocarriers or applied materials in many cases, thus the choice of these nanocarriers or applied materials should be taken into full consideration. For example, if we use highly safe, effective and precise nanocarriers to deliver cytokines, safety, effectiveness, and precision would be guaranteed. Radiation, magnetic forces, light, and some other external stimuli are very effective tools, but we should consider the tolerance threshold of human body. Over strong stimulus can cause serious side effects, too weak one may not be effective. Thus, we should look for more suitable nanocarriers or applied materials or moderate stimulation to improve specificity, safety, and effectiveness. Finally, appropriate methods are selected based on the type of disease tissues and pathological features.

To overcome vascular obstacles for improved nanotheranostic outcomes, the in‐depth understanding of interaction mechanism between nanotheranostic agents and blood vessels should be further sought. Meanwhile, we also need to put more effort into scaled‐up synthesis, long‐term assessment of toxicity and establishment of regulatory protocols of nanotheranostic agents. Additionally, we first should stratify patients and recommend a careful consideration of the individual characteristics and pathological location of the disease tissues to rationally combine several strategies mentioned above to create a tailor‐designed strategy of overcoming vascular barrier. The advantage of this combined strategy is that every strategy progressively plays different role in different position and times in vivo to maximize the nanotheranostic outcome and minimize side‐effect. It will promote the clinical transformation of nanomedicines. More importantly, we should develop new theranostic strategies that does not require nanotheranostic agents to pass through the vascular barriers to reach the targeted disease cells or tissues. For example, disease immunotherapy agents can generate a universal immune response in whole body to clear the distal disease cells or exogenous intruders. In the future, more practical and effective strategies are expected to be applied into clinical translational for improved nanomedicine theranostics with new discoveries in nanotechnology science and evolutionary understanding of the vascular barriers.

## Conflict of Interest

The authors declare no conflict of interest.
